# The impact of ECPELLA on haemodynamics and global oxygen delivery: a comprehensive simulation of biventricular failure

**DOI:** 10.1186/s40635-024-00599-7

**Published:** 2024-02-16

**Authors:** Hiroki Matsushita, Keita Saku, Takuya Nishikawa, Shohei Yokota, Kei Sato, Hidetaka Morita, Yuki Yoshida, Masafumi Fukumitsu, Kazunori Uemura, Toru Kawada, Ken Yamaura

**Affiliations:** 1 Department of Cardiovascular Dynamics, National Cerebral and Cardiovascular Center Research Institute, 6-1 Kishibe-Shimmachi, Suita, Osaka 564-8565 Japan; 2 Department of Research Promotion and Management, National Cerebral and Cardiovascular Center Research Institute, Suita, Japan; 3 NTTR-NCVC Bio Digital Twin Centre, National Cerebral and Cardiovascular Center Research Institute, Suita, Japan; 4 Department of Anesthesiology and Critical Care Medicine, Graduate School of Medical Sciences, Kyushu University, Fukuoka, Japan

**Keywords:** Simulation, Cardiogenic shock, VA-ECMO, Impella, ECPELLA, Haemodynamics, Oxygen delivery

## Abstract

**Background:**

ECPELLA, a combination of veno-arterial (VA) extracorporeal membrane oxygenation (ECMO) and Impella, a percutaneous left ventricular (LV) assist device, has emerged as a novel therapeutic option in patients with severe cardiogenic shock (CS). Since multiple cardiovascular and pump factors influence the haemodynamic effects of ECPELLA, optimising ECPELLA management remains challenging. In this study, we conducted a comprehensive simulation study of ECPELLA haemodynamics. We also simulated global oxygen delivery (DO_2_) under ECPELLA in severe CS and acute respiratory failure as a first step to incorporate global DO_2_ into our developed cardiovascular simulation.

**Methods and results:**

Both the systemic and pulmonary circulations were modelled using a 5-element resistance‒capacitance network. The four ventricles were represented by time-varying elastances with unidirectional valves. In the scenarios of severe LV dysfunction, biventricular dysfunction with normal pulmonary vascular resistance (PVR, 0.8 Wood units), and biventricular dysfunction with high PVR (6.0 Wood units), we compared the changes in haemodynamics, pressure–volume relationship (PV loop), and global DO_2_ under different VA-ECMO flows and Impella support levels.

**Results:**

In the simulation, ECPELLA improved total systemic flow with a minimising biventricular pressure–volume loop, indicating biventricular unloading in normal PVR conditions. Meanwhile, increased Impella support level in high PVR conditions rendered the LV–PV loop smaller and induced LV suction in ECPELLA support conditions. The general trend of global DO_2_ was followed by the changes in total systemic flow. The addition of veno-venous ECMO (VV-ECMO) augmented the global DO_2_ increment under ECPELLA total support conditions.

**Conclusions:**

The optimal ECPELLA support increased total systemic flow and achieved both biventricular unloading. The VV-ECMO effectively improves global DO_2_ in total ECPELLA support conditions.

**Supplementary Information:**

The online version contains supplementary material available at 10.1186/s40635-024-00599-7.

## Background

Despite notable advancements in cardiovascular intensive care, cardiogenic shock (CS) remains associated with a high mortality rate [1]. Veno-arterial extracorporeal membrane oxygenation (VA-ECMO) has revolutionised the management of CS, providing essential global oxygen delivery (DO_2_) and haemodynamic support to patients with complicated haemodynamics. However, VA-ECMO can also increase left ventricular (LV) afterload, potentially worsening LV dysfunction and pulmonary oedema [2]. Recently, ECPELLA, the combination of VA-ECMO and Impella, a percutaneous LV assist device (Abiomed Inc., Danvers, MA, USA), has emerged as a novel therapeutic option to address the limitation of VA-ECMO alone strategy [3].

ECPELLA effectively unloads the LV and augments systemic blood flow. However, the haemodynamic effects of ECPELLA are complex and influenced by a multitude of factors, including the underlying cardiovascular and lung condition and the VA-ECMO flow and Impella support level settings. Thus, optimising ECPELLA management remains challenging [4].

Moreover, 80% of patients with CS develop acute respiratory failure (ARF) [5]. In some cases of severe CS with ARF, impaired oxygenation in the lung requires veno-venous ECMO (VV-ECMO) in addition to other mechanical circulatory support (MCS) [6]. Multi-optional MCS strategies may save the patients with severe CS. However, difficulty in accumulating case experience and case-dependent haemodynamic and respiratory alterations limit a comprehensive understanding of these treatments.

We conducted a simulation study of ECPELLA haemodynamic management to understand ECPELLA haemodynamics comprehensively, leading to optimal ECPELLA management. We have previously reported the haemodynamic mechanisms of several MCSs using an electrical cardiovascular model [7]. Simulations allow us to evaluate and visualise the dynamic changes of right atrial pressure (RAP), left atrial pressure (LAP), total systemic flow, and biventricular pressure–volume loop (PV loop) at varying VA-ECMO flow, Impella support level, and degree of biventricular function, which are difficult to assess in clinical practice. We also simulated global DO_2_ in severe CS and ARF with ECPELLA support as a first step to incorporate global DO_2_ management into our developed cardiovascular simulation.

## Methods

### Electrical model

**Fig. 1:**
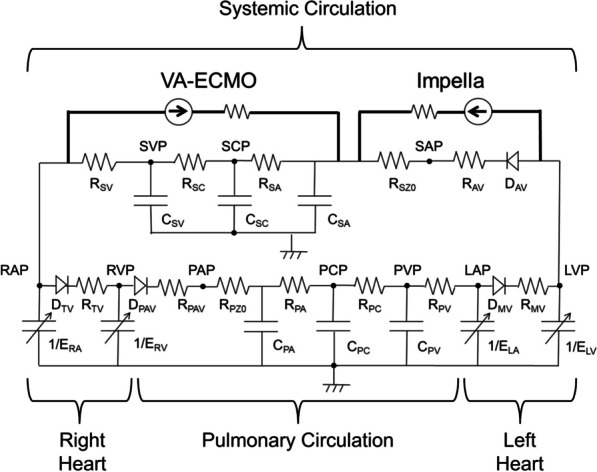
Circuit diagram of the cardiovascular simulation model. *VA-ECMO* veno-arterial extracorporeal membrane oxygenation, *LAP* left atrial pressure, *LVP* left ventricular pressure, *SAP* systemic arterial pressure, *SCP* systemic capillary pressure, *SVP* systemic venous pressure, *RAP* right atrial pressure, *RVP* right ventricular pressure, *PAP* pulmonary artery pressure, *PCP* pulmonary capillary pressure, *PVP* pulmonary venous pressure, *E*_*LA*_ time-varying elastance of left atrium, *D*_*MV*_ mitral valve, *R*_*MV*_ resistance of mitral valve, *E*_*LV*_ time-varying elastance of left ventricle, *D*_*AV*_ aortic valve, *R*_*AV*_ resistance of aortic valve, *R*_*SZ0*_ characteristic impedance of systemic circulation, *C*_*SA*_ compliance of systemic circulation, *R*_*SA*_ resistance of systemic artery, *C*_*SC*_ compliance of systemic capillary vessels, *R*_*SC*_ resistance of systemic capillary vessels, *C*_*SV*_ compliance of systemic vein, *R*_*SV*_ resistance of systemic vein, *E*_*RA*_ time-varying elastance of right atrium, *D*_*TV*_ tricuspid valve, *R*_*TV*_ resistance of tricuspid valve, *E*_*RV*_ time-varying elastance of right ventricle, *D*_*PAV*_ pulmonary artery valve, *R*_*PAV*_ resistance of pulmonary artery valve, *R*_*PZ0*_ characteristic impedance of pulmonary circulation, *C*_*PA*_ compliance of pulmonary artery, *R*_*PA*_ resistance of pulmonary artery, *C*_*PC*_ compliance of pulmonary capillary vessels, *R*_*PC*_ resistance of pulmonary capillary vessels, *C*_*PV*_ compliance of pulmonary vein, *R*_*PV*_ resistance of pulmonary vein

We examined the impact of MCS combination therapies on haemodynamics and oxygen delivery in silico using a 5-element cardiovascular model (Fig. 1). We simulated the dynamic cardiovascular system using Simulink^®^ (Mathworks, Massachusetts, USA). The systemic and pulmonary circulations were modelled using the 5-element resistance‒capacitance network model. We approximated four intracardiac valves as unidirectional valves, and the flow rate was determined by the pressure gradient between pre- and post-valve compartments and the valve orifice area based on Bernoulli's theorem [8]. In the four cardiac chambers, contractility and relaxation were represented by time-varying elastance [9], and stiffness was expressed by the end-diastolic pressure–volume relation [10]. VA-ECMO was designed to continuously remove blood from systemic veins and return to the systemic arteries at continuous flows. Impella was designed to withdraw blood from the LV and continuously return blood to systemic arteries by the axial pump. We modelled the pump performance of Impella based on the head‒capacity (H‒Q) curve described in a previous report [11] and the company's published product manual (Instructions for Use and Clinical Reference Manual of Impella CP). The flow rate of Impella was determined by the Impella rotational speed (P0‒P9) and the pressure gradient between the systemic artery and LV (Additional files 1 and 2). The approximate H–Q curves (P0–P9) correlate significantly (*p* < 0.05, Spearman's rank correlation coefficient) with the published H–Q curves (Additional file 3).

### Setting of parameters and outcomes

To simulate left ventricular failure (LVF) and biventricular failure (BVF) without any mechanical circulatory support, we adjusted several parameters as follows: LV end-systolic elastance (E_es_) was set at 0.4 mmHg/ml; right ventricular (RV) E_es_ was varied for 0.2 and 0.5 mmHg/mL; heart rate was set to 80 beats per minute (bpm) with reference to several clinical studies of CS [12–14], and kept constant for simplicity of simulation, and systemic vascular resistance (SVR) was set at 11.7 Wood units (WU). Previous studies have reported normal values of 1.6 mmHg/ml for LV‒E_es_ and 0.44 mmHg/ml for RV‒E_es_, although the values may vary depending on species and the measurement method [15, 16]. Each E_es_ in this study referenced studies of cardiogenic shock models caused by acute myocardial infarction or acute pulmonary artery thrombosis, showing that contractility is reduced by 34‒51% from baseline [17, 18]. To simulate the conditions of healthy physiology and pulmonary hypertension (PH), we modulated the total pulmonary vascular resistance (PVR), setting it to 0.8 WU for healthy and 6.0 WU for PH. Pulmonary vascular impedance (Zc) was adjusted according to changes in PVR as reported in previous studies [19], while systemic parameters and other physiological parameters were fixed values based on data from healthy subjects (Additional file 1).

**Fig. 2:**
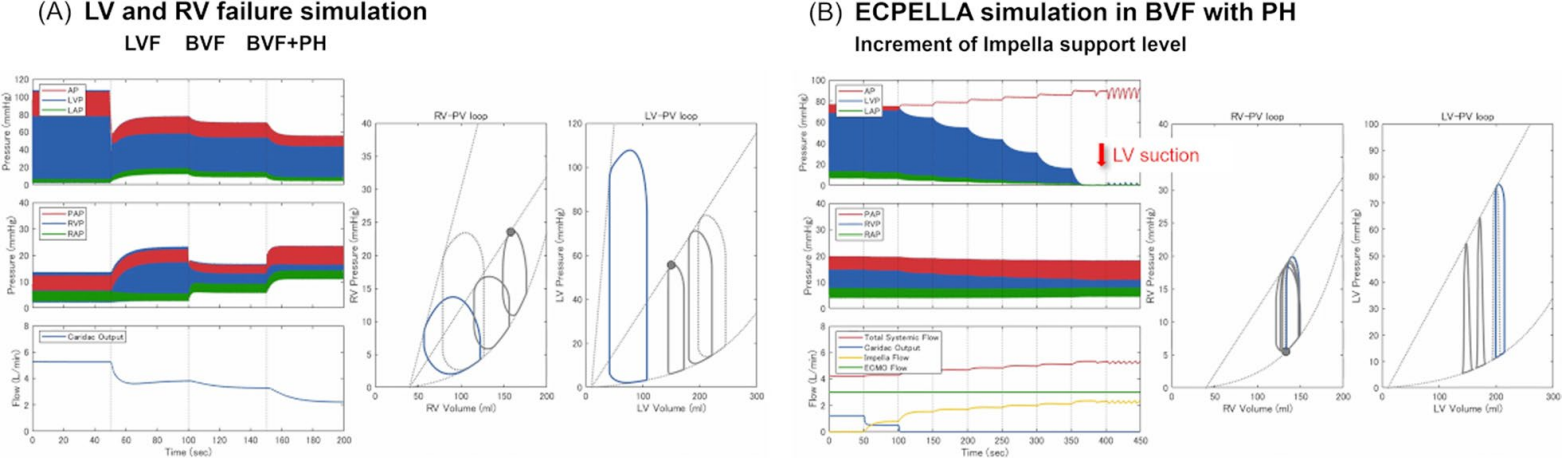
**A** Representative plots from one cardiovascular simulation with changing cardiovascular conditions. Our simulation can capture complicated haemodynamic changes and generate biventricular pressure–volume loops for each heartbeat. The plot draws haemodynamic changes from the normal condition to LVF, BVF, and BVF with PH. *LVF* left ventricular failure, *BVF* biventricular failure, *PH* pulmonary hypertension, *AP* arterial pressure, *LVP* left ventricle pressure, *LAP* left atrial pressure, *PAP* pulmonary artery pressure, *RVP* right ventricular pressure, *RAP* right atrial pressure, *PV loop* pressure–volume loop. **B** Representative plots from one cardiovascular simulation with changing VA-ECMO flows and Impella support levels. In biventricular failure and high pulmonary vascular resistance, the combination therapy of VA-ECMO and Impella (ECPELLA) increased AP and total systemic flow while decreasing LVP and inducing LV suction with high-flow Impella support. *VA-ECMO* veno-arterial extracorporeal membrane oxygenation, *AP* arterial pressure, *LVP* left ventricular pressure, *LAP* left atrial pressure, *PAP* pulmonary artery pressure, *RVP* right ventricular pressure, *RAP* right atrial pressure, *PV loop* pressure–volume loop

We constructed PV loops from the dynamic data obtained over time by conducting a simulation for each condition (Fig. 2). We calculated the pressure–volume area (PVA) [20] by integrating the area enclosed by the end-systolic pressure–volume relationship (ESPVR), the end-diastolic pressure‒volume relationship (EDPVR), and the PV loop (Additional file 4). Additionally, we determined stroke work (SW) [20] as the area within the PV loop for a single cardiac cycle. We compared the changes in RAP and LAP, total systemic flow, PVA, and SW across three pathological conditions: left LVF, BVF, and BVF concomitant with PH. These conditions were examined under different VA-ECMO flows and Impella support levels.

Furthermore, we evaluate the effect of multiple MCS on global DO_2_. Global DO_2_ is determined by cardiac output (CO), haemoglobin concentration, and arterial oxygen saturation (SaO_2_) [21]. Using ECPELLA support, Impella delivers blood oxygenated by the patient's lungs, and VA-ECMO supplies blood highly oxygenated by artificial lungs. Therefore, global DO_2_ under ECPELLA support is represented by the following equation:

$${\text{DO}}_{2} = 1.34 \times 10 \times {\text{Hb}} \times {\text{SaO}}_{2} \times ({\text{CO}} + {\text{Impella flow}} ) + 1.34 \times 10 \times {\text{Hb}} \times 1.0 \times {\text{VA-ECMO flow}},$$

where Hb (g/dL) is the haemoglobin concentration, SaO_2_ (–) is arterial oxygen saturation, and CO (L/min) is cardiac output.

In clinical practice, excessive blood withdrawal by Impella can lead to a significant decrease in LV volume, resulting in "LV suction" [22]. In this study, LV suction was defined as LAP reaching 0 mmHg. We adjusted the simulation to introduce resistance to the Impella flow when LAP fell below 0, reducing flow.

### Protocols

#### Protocol 1: impact of ECPELLA on LVF haemodynamics

We examined the effect of VA-ECMO flow and Impella support level on haemodynamics in LV dysfunction. VA-ECMO varied from 0 to 5 L/min in 0.5 L/min steps, and Impella also varied in each support stepwise from P0 to P9. LV‒*E*_es_ was set at 0.4 mmHg/ml. RV systolic function and PVR were fixed at normal levels (RV‒*E*_es_, 0.5 mmHg/mL, PVR, 0.8 WU).

#### Protocol 2: impact of ECPELLA on BVF haemodynamics

We investigated the impact of VA-ECMO flows and Impella support levels on haemodynamics in biventricular dysfunction. VA-ECMO varied from 0 to 5 L/min in 0.5 L/min steps, and Impella also varied in each support stepwise from P0 to P9. LV‒*E*_es_ and RV‒*E*_es_ were set at 0.4 and 0.2 mmHg/mL, respectively. PVR was fixed at normal levels (PVR, 0.8 WU).

#### Protocol 3: impact of ECPELLA on BVF with PH haemodynamics

We investigated the impact of VA-ECMO flows and Impella support levels on haemodynamics in biventricular dysfunction with pulmonary hypertension. VA-ECMO varied from 0 to 5 L/min in 0.5 L/min steps, and Impella also varied in each support stepwise from P0 to P9. LV‒*E*_es_ and RV‒*E*_es_ were set at 0.4 and 0.2 mmHg/mL, respectively. PVR was set at 6.0 WU.

#### Protocol 4: impact of ECEPLLA with/without VV-ECMO on global DO_2_

We performed haemodynamic simulations in the ECPELLA support condition with VV-ECMO. To simulate typical clinical scenarios, haemoglobin concentration was set at 10 g/dL, which refers to the common clinical setting as shown in the recent VV-ECMO study [23], with a target SaO_2_ of 80% as recommended by the Extracorporeal Life Support Organization [24]. To simulate severe ARF with hypoxemia, SaO_2_ was set at 40% for the native lung and increased to 80% by adding VV-ECMO support. The maximum VA-ECMO flow rate was set at 4 L/min, considering that an increase in venous outflow can lead to a reduction in arterial outflow due to the inherent limitations of venous circulation in VAV-ECMO support [25]. We also validated two different patterns of PVR with pulmonary oedema. LV‒*E*_es_ and RV‒*E*_es_ were set at 0.4 and 0.2 mmHg/mL, respectively. PVR were set at 0.8 WU (normal PVR) and 6.0 WU (PH).

### Data analysis

The fixed step size (fundamental sampling time) in this simulation was set at 0.2 ms, and we performed calculations for 550 s in each simulation. For the first 100 s, haemodynamic simulations were conducted without Impella support. Then, the Impella support level was increased gradually every 50 s, and stable haemodynamic values were extracted 2 s before the next alteration when the time series data reached a steady state (Additional file 5) [26].

## Results

### Protocol 1: impact of ECPELLA on LVF haemodynamics

**Fig. 3:**
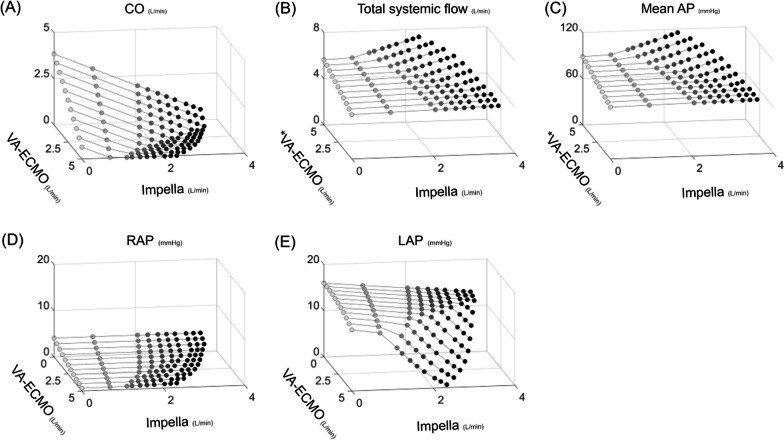
Impact of ECPELLA on LVF haemodynamics. Changes in CO (**A**), total systemic flow (**B**), mean AP (**C**), RAP (**D**), and LAP (**E**) are shown. Each simulation consists of a series of 10 plots (Impella support at P0 ➝ P1 ➝・・・➝P9) corresponding to a single varied setting, from which 11 sets of data (VA-ECMO flow at 0➝0.5➝・・・➝5.0 L/min) were used to construct the plots. An increase in VA-ECMO flow decreased CO and increased total systemic flow and mean AP. The addition of Impella decreased CO and increased total systemic flow and mean AP, while maintaining RAP and reducing LAP. Under total ECPELLA support, i.e. zero CO, an increase in Impella support level effectively increased total systemic flow and mean AP, and markedly decreased LAP. *LVF* left ventricular failure, *VA-ECMO* veno-arterial extracorporeal membrane oxygenation, *CO* cardiac output, *AP* arterial pressure, *RAP* right atrial pressure, *LAP* left atrial pressure

**Fig. 4:**
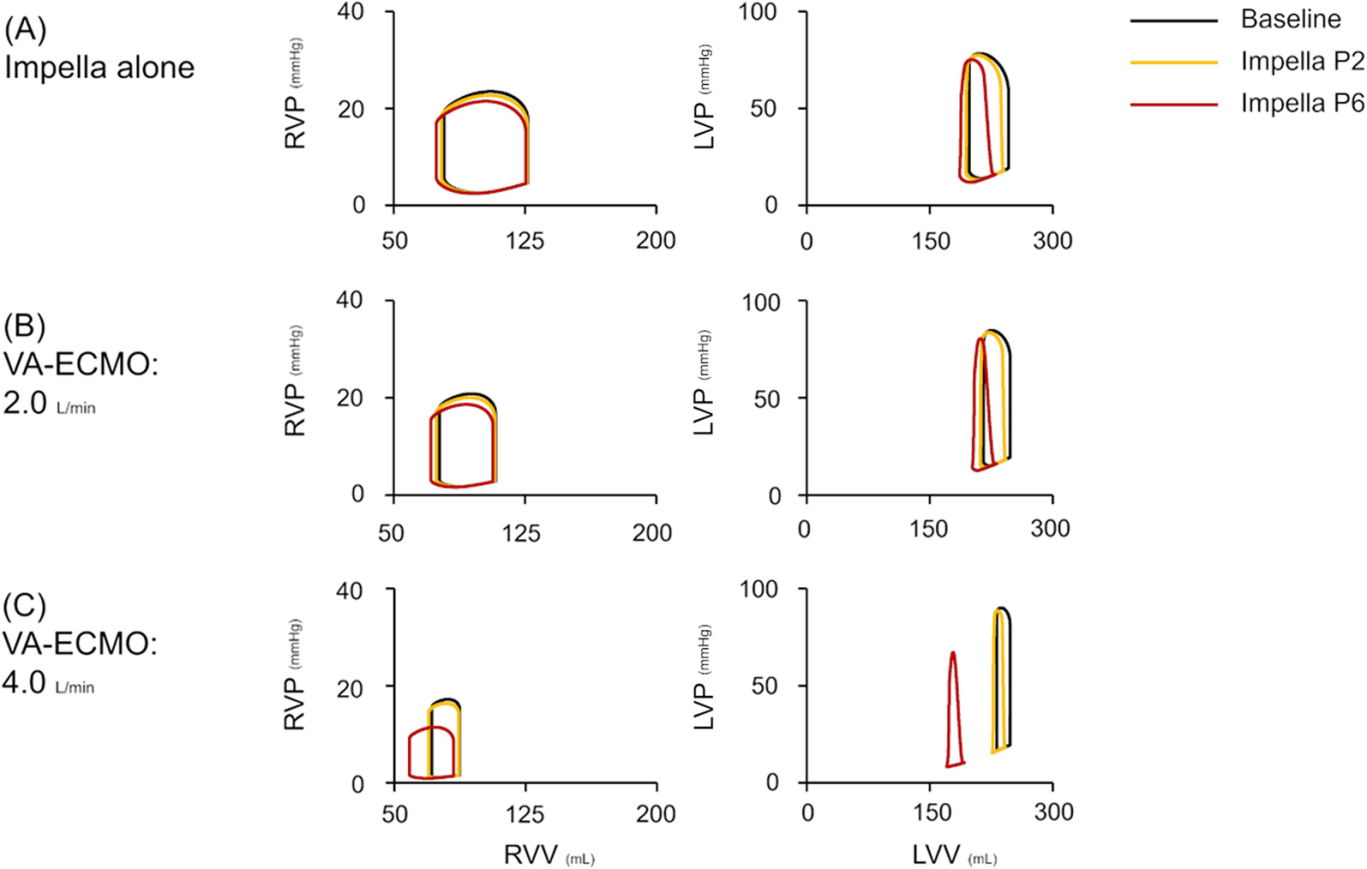
Impact of ECPELLA on right and left ventricular PV loops on LVF haemodynamics. Three conditions of MCS are shown: Impella alone (**A**), 2.0 mL/min of VA-ECMO with Impella (**B**), and 4.0 mL/min of VA-ECMO with Impella (**C**). Different conditions are represented by various colours (black line: baseline, yellow: supported with Impella P2, red: Impella P6). In the RV-PV loop, both VA-ECMO and Impella rendered the RV-PV loop left downward in each ECPELLA support condition. In the LV-PV loop, VA-ECMO shifted the LV-PV loop downward to the left only in the higher Impella support conditions (P6). *PV loop* pressure–volume loop, *LVF* left ventricular failure, *VA-ECMO* veno-arterial extracorporeal membrane oxygenation, *RVP* right ventricular pressure, *RVV* right ventricular volume, *LVP* left ventricular pressure, *LVV* left ventricular volume

As shown in Figs. 3 and 4, an increase in VA-ECMO flow decreased CO and increased total systemic flow and mean AP. The addition of Impella decreased CO and increased total systemic flow and mean AP while maintaining RAP and reducing LAP. Under total ECPELLA support, i.e. zero CO and ECPELLA-dependent circulation, an increase in Impella support level effectively increased total systemic flow and mean AP and markedly decreased LAP. PV loop analyses are shown in Fig. 4 and Additional file 6. In the RV-PV loop, both VA-ECMO and Impella rendered the RV-PV loop left downward and decreased RV-PVA in each ECPELLA support condition. In the LV–PV loop, VA-ECMO shifted the LV–PV loop downward to the left only in higher Impella support conditions (P6). The degree of LV–PV loop shifting and LV-PVA reduction by Impella were augmented under VA-ECMO support conditions.

### Protocol 2: impact of ECPELLA on BVF haemodynamics

**Fig. 5:**
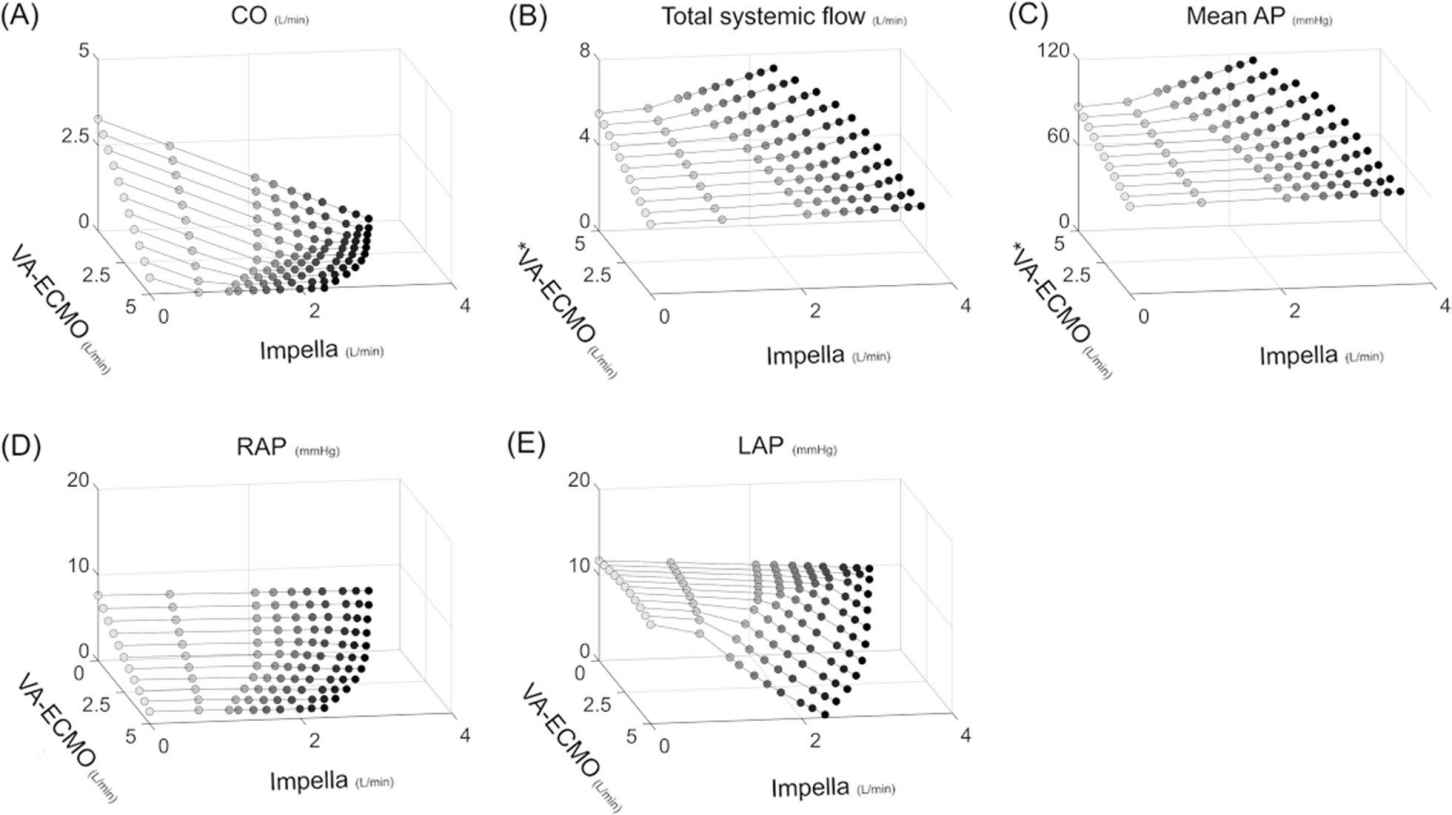
Impact of ECPELLA on BVF haemodynamics. Changes in CO (**A**), total systemic flow (**B**), mean AP (**C**), RAP (**D**), and LAP (**E**) are shown. An asterisk (*) indicates that the corresponding axis has been inverted to clarify or highlight specific relationships. The general trends were the same as in protocol 1. Under total ECPELLA support, an increase in Impella support level effectively increased total systemic flow and mean AP, and markedly decreased LAP. *BVF* biventricular failure, *VA-ECMO* veno-arterial extracorporeal membrane oxygenation, *CO* cardiac output, *AP* arterial pressure, *RAP* right atrial pressure, *LAP* left atrial pressure

**Fig. 6:**
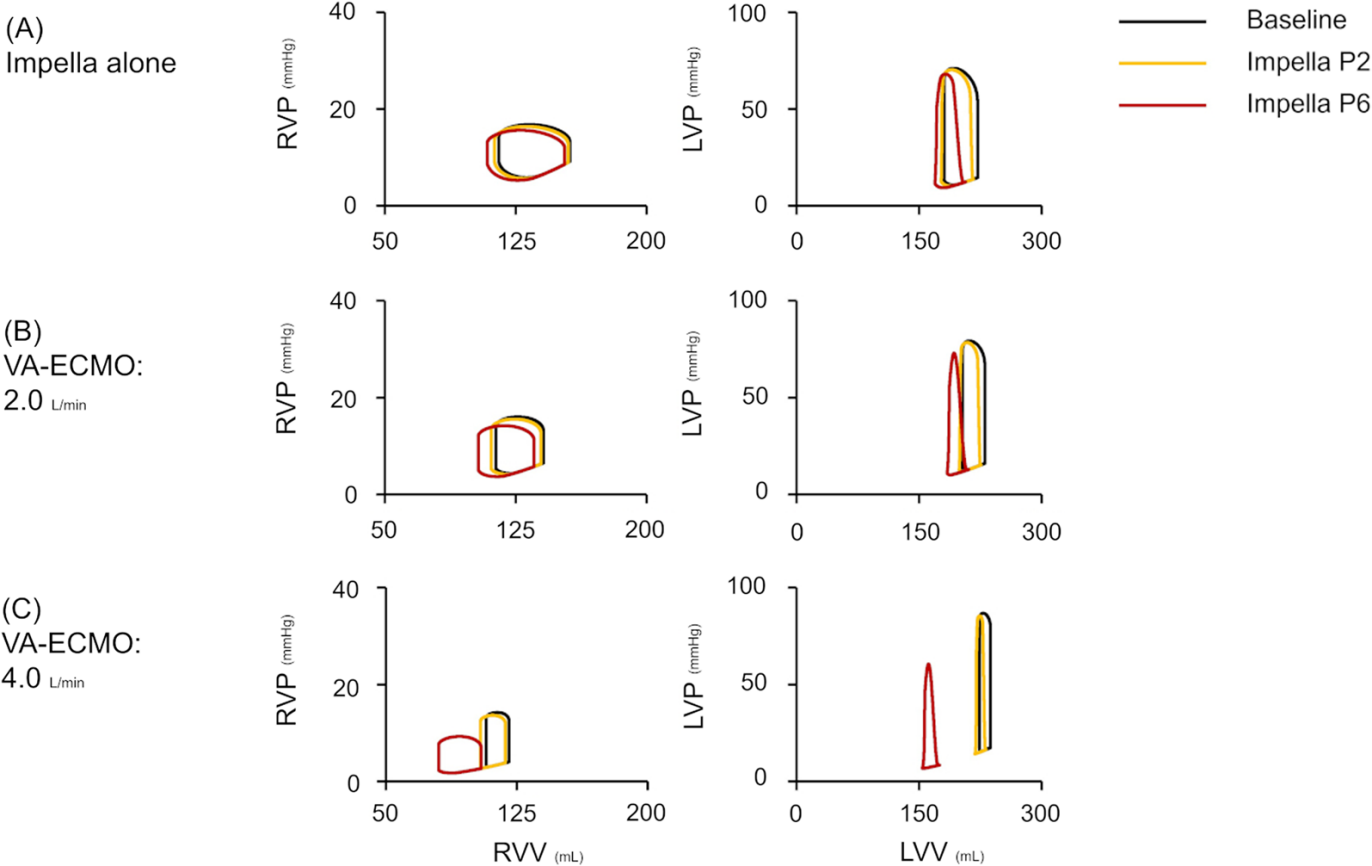
Impact of ECPELLA on the right and left ventricular PV loops on BVF haemodynamics. Three conditions of MCS are shown: Impella alone (**A**), 2.0 mL/min of VA-ECMO with Impella (**B**), and 4.0 mL/min of VA-ECMO with Impella (**C**). Different conditions are represented by various colours (black line: baseline, yellow: supported with Impella P2, red: Impella P6). The PV loop changes were similar to the results of protocol 1. In higher VA-ECMO conditions, the increase in Impella support level markedly shifted both the RV- and LV-PV loops downward to the left. *PV loop* pressure–volume loop, *BVF* biventricular failure, *MCS* mechanical circulatory support, *VA-ECMO* veno-arterial extracorporeal membrane oxygenation, *RVP* right ventricular pressure, *RVV* right ventricular volume, *LVP* left ventricular pressure, *LVV* left ventricular volume

Figures 5 and 6 represent the haemodynamic changes with ECPELLA in BVF conditions. The general trends were the same as in protocol 1. Under total ECPELLA support, an increase in Impella support level effectively increased total systemic flow and mean AP and markedly decreased LAP. PV loop analyses are shown in Fig. 6 and Additional file 6. The PV loop changes were similar to the results of protocol 1. In higher VA-ECMO conditions, the increase in Impella support level markedly shifted both the RV- and LV–PV loops downward to the left.

### Protocol 3: impact of ECPELLA on BVF with PH haemodynamics

**Fig. 7:**
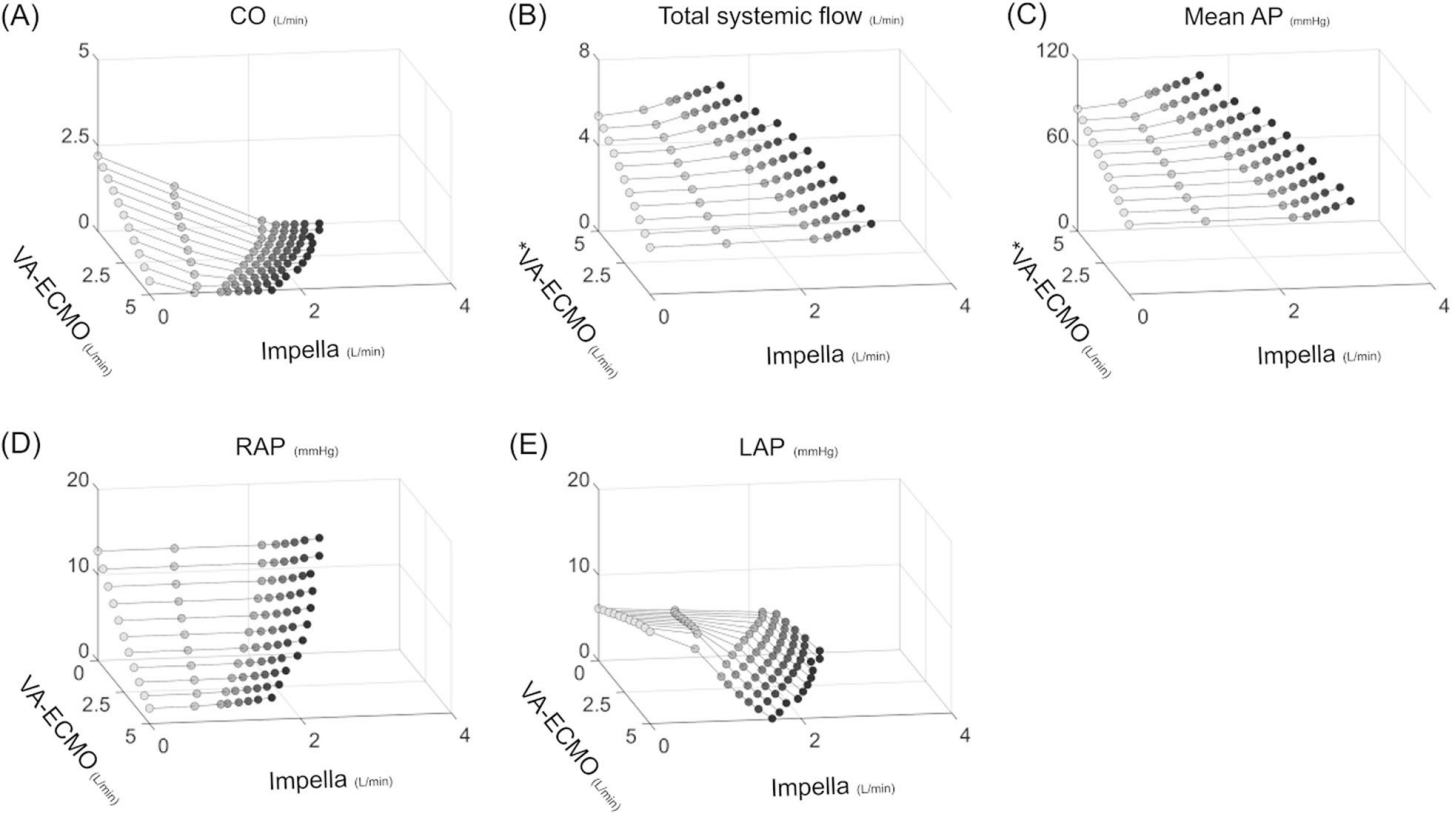
Impact of ECPELLA on BVF with PH haemodynamics. Changes in CO (**A**), total systemic flow (**B**), mean AP (**C**), RAP (**D**), and LAP (**E**) are shown. An asterisk (*) indicates that the corresponding axis has been inverted to clarify or highlight specific relationships. Since the PH further reduced LV filling, an increase in Impella support level excessively reduced LAP, induced LV suction, and limited the Impella support level (less than 1.6 L/min) in each VA-ECMO flow condition. *BVF* biventricular failure, *PH* pulmonary hypertension, *VA-ECMO* veno-arterial extracorporeal membrane oxygenation, *CO* cardiac output, *AP* arterial pressure, *RAP* right atrial pressure, *LAP* left atrial pressure

**Fig. 8:**
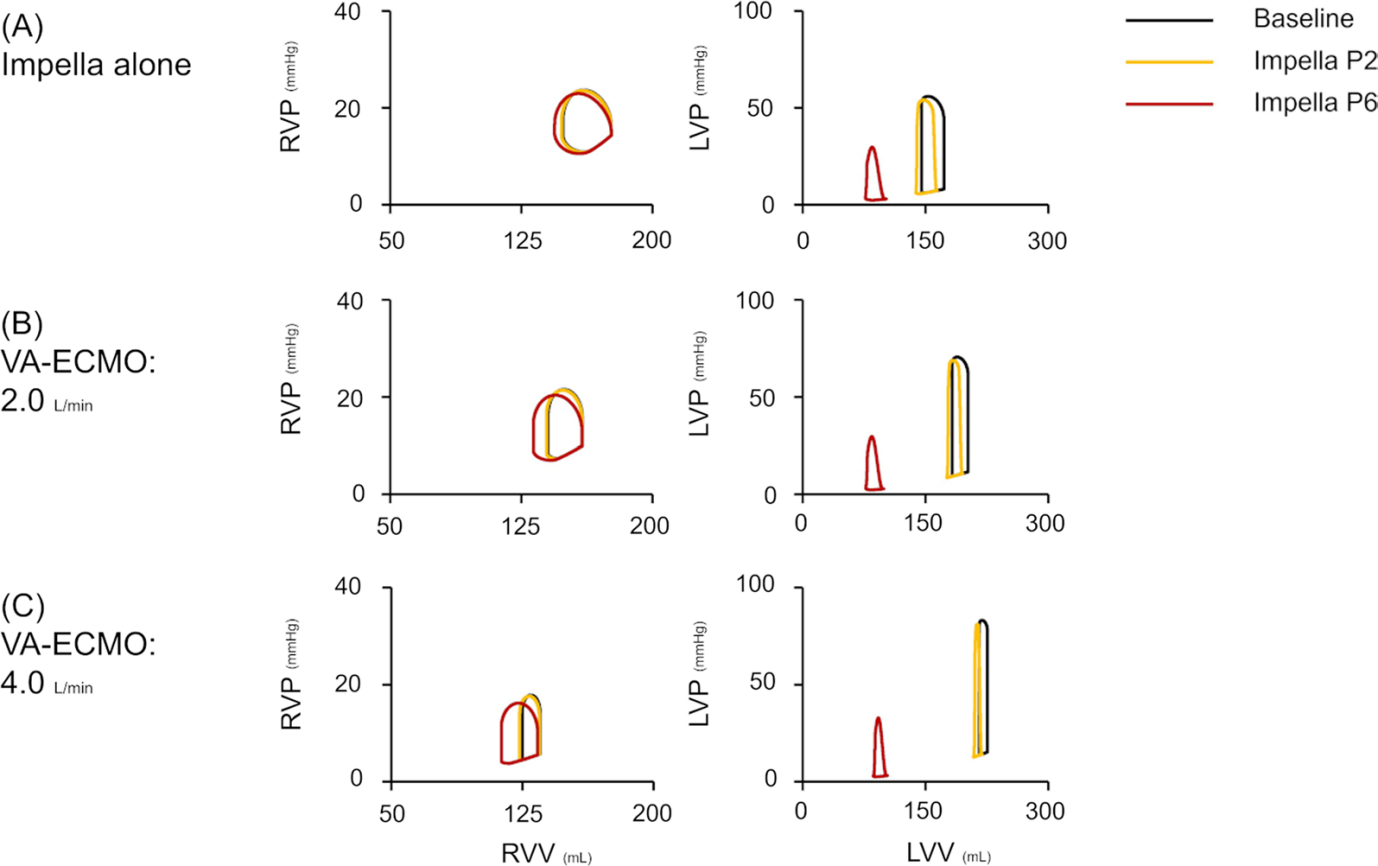
Impact of ECPELLA on right and left ventricular PV loops on BVF with PH haemodynamics. Three conditions of MCS are shown: Impella alone (**A**), 2.0 mL/min of VA-ECMO with Impella (**B**), and 4.0 mL/min of VA-ECMO with Impella (**C**). Different conditions are represented by various colours (black line: Baseline, yellow: supported with Impella P2, red: Impella P6). In the BVF with PH under ECPELLA support, an increase in Impella support level widened the RV-PV loop but did not change RV-EDV compared to the BVF condition (protocol 2). Higher Impella support level strikingly minimised the LV-PV loop in each VA-ECMO flow condition. *PV loop* pressure–volume loop, *BVF* biventricular failure, *PH* pulmonary hypertension, *MCS* mechanical circulatory support, *VA-ECMO* veno-arterial extracorporeal membrane oxygenation, *RVP* right ventricular pressure, *RVV* right ventricular volume, *LVP* left ventricular pressure, *LVV* left ventricular volume

Figures 7 and 8 represent the haemodynamic changes with ECPELLA in BVF with PH. Since the PH further reduced LV filling, an increase in Impella support level excessively reduced LAP, induced LV suction, and limited the Impella support level (less than 1.6 L/min) in each VA-ECMO flow condition. PV loop analyses are shown in Fig. 8 and Additional file 6. In the BVF with PH under ECPELLA support, an increase in Impella support level widened the RV-PV loop but did not change RV-EDV compared to the BVF condition (protocol 2). Higher Impella support level strikingly minimised the LV-PV loop in each VA-ECMO flow condition.

#### Protocol 4: impact of ECEPLLA on global DO_2_ in the absence and presence of VV-ECMO support

**Fig. 9:**
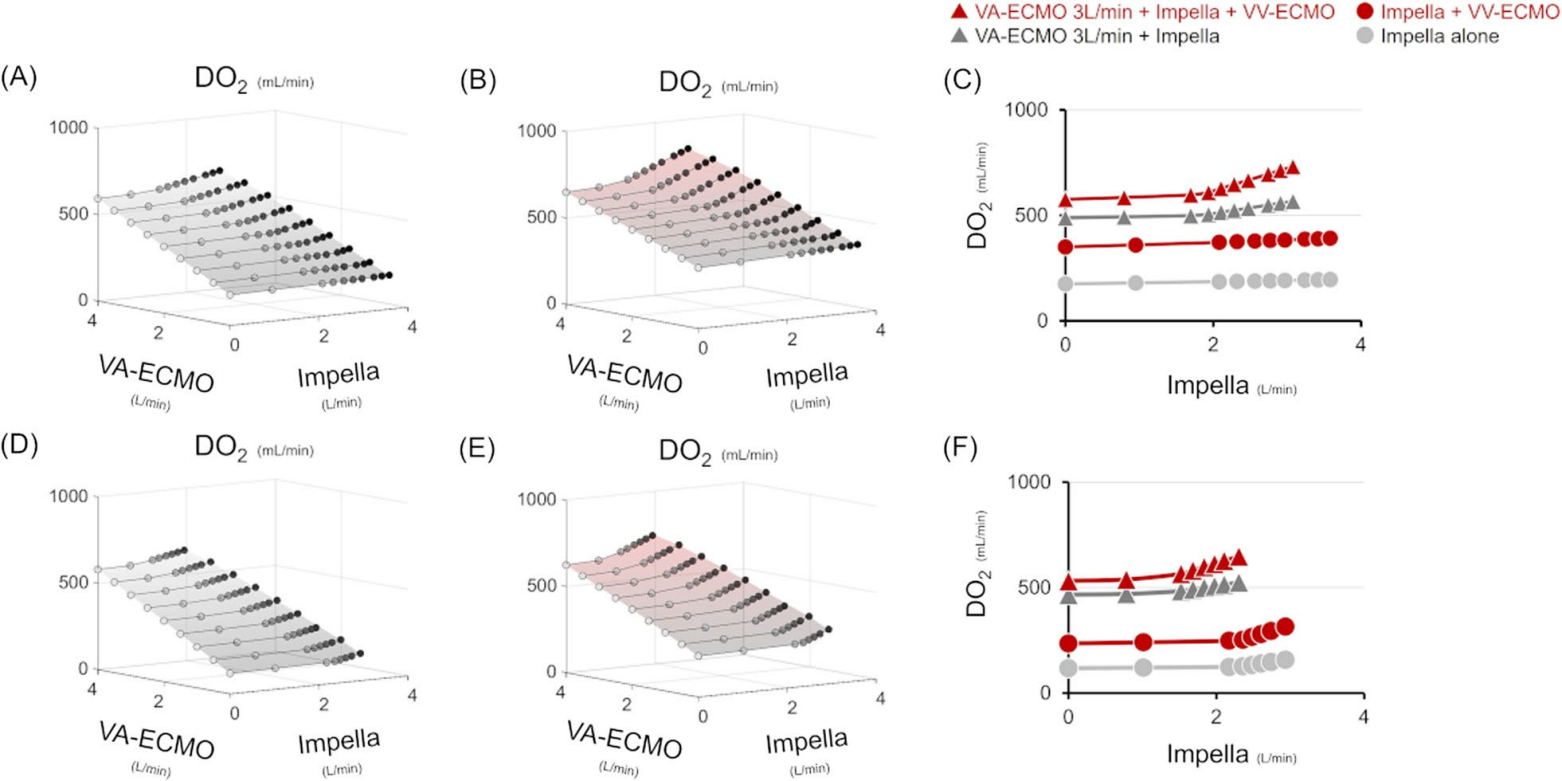
Impact of ECPELLA on global DO_2_ with and without VV-ECMO Support. Upper panels represent the changes of global DO_2_ without (SaO_2_: 40%, **A**) and with (SaO_2_: 80%, **B**) VV-ECMO, and the comparison between the two conditions (**C**) in BVF supported by ECPELLA. Haemoglobin concentration was set at 10 g/dL. Lower panels represent the changes of global DO_2_ without (SaO_2_: 40%, **D**) and with (SaO_2_: 80%, **E**) VV-ECMO, and the comparison between the two conditions (**F**) in BVF with PH supported by ECPELLA. Different conditions are represented by different colours (light grey line: Impella alone, dark grey line: VA-ECMO with Impella, red line: with VV-ECMO) and markers (round: Impella, triangle: VA-ECMO with Impella). The changes in global DO_2_ closely correspond to the changes in systemic flow in the ECPELLA support condition. The elevation of SaO_2_, by using VV-ECMO, increased global DO_2_ and augmented the effect of Impella support level on global DO_2_ elevation. *DO*_*2*_ oxygen delivery, *VV-ECMO* veno-venous membrane oxygenation, *VA-ECMO* veno-arterial extracorporeal membrane oxygenation, *SaO*_*2*_ arterial oxygen saturation

Figure 9 represents global DO_2_ simulations with low SaO_2_ due to severe ARF. The changes in global DO_2_ closely correspond to the changes in systemic flow in the ECPELLA support condition. Thus, the total support of ECPELLA effectively increased global DO_2_. The elevation of SaO_2_, using VV-ECMO, increased global DO_2_ and augmented the effect of Impella support level on global DO_2_ elevation. In the high PVR condition, the increase in global DO_2_ with ECPELLA support was limited, reflecting the restricted total systemic flow due to LV suction.

## Discussion

In this study, we used cardiovascular simulation to demonstrate the impact of VA-ECMO and Impella on haemodynamics in various cardiovascular conditions and the effect of VV-ECMO on global DO_2_ in BVF with severe ARF. The major findings of this study were as follows: (1) VA-ECMO increases total systemic flow depending on the flow rate. Meanwhile, Impella effectively increases total systemic flow in total ECPELLA support conditions. (2) ECPELLA can provide RV and LV unloading, while an appropriate increase in Impella support level may induce LV suction, especially in high PVR conditions. (3) The addition of VV-ECMO enhances the global DO_2_ augmentation effect of Impella in BVF with severe ARF.

### The impact of ECPELLA on total systemic flow

**Fig. 10:**
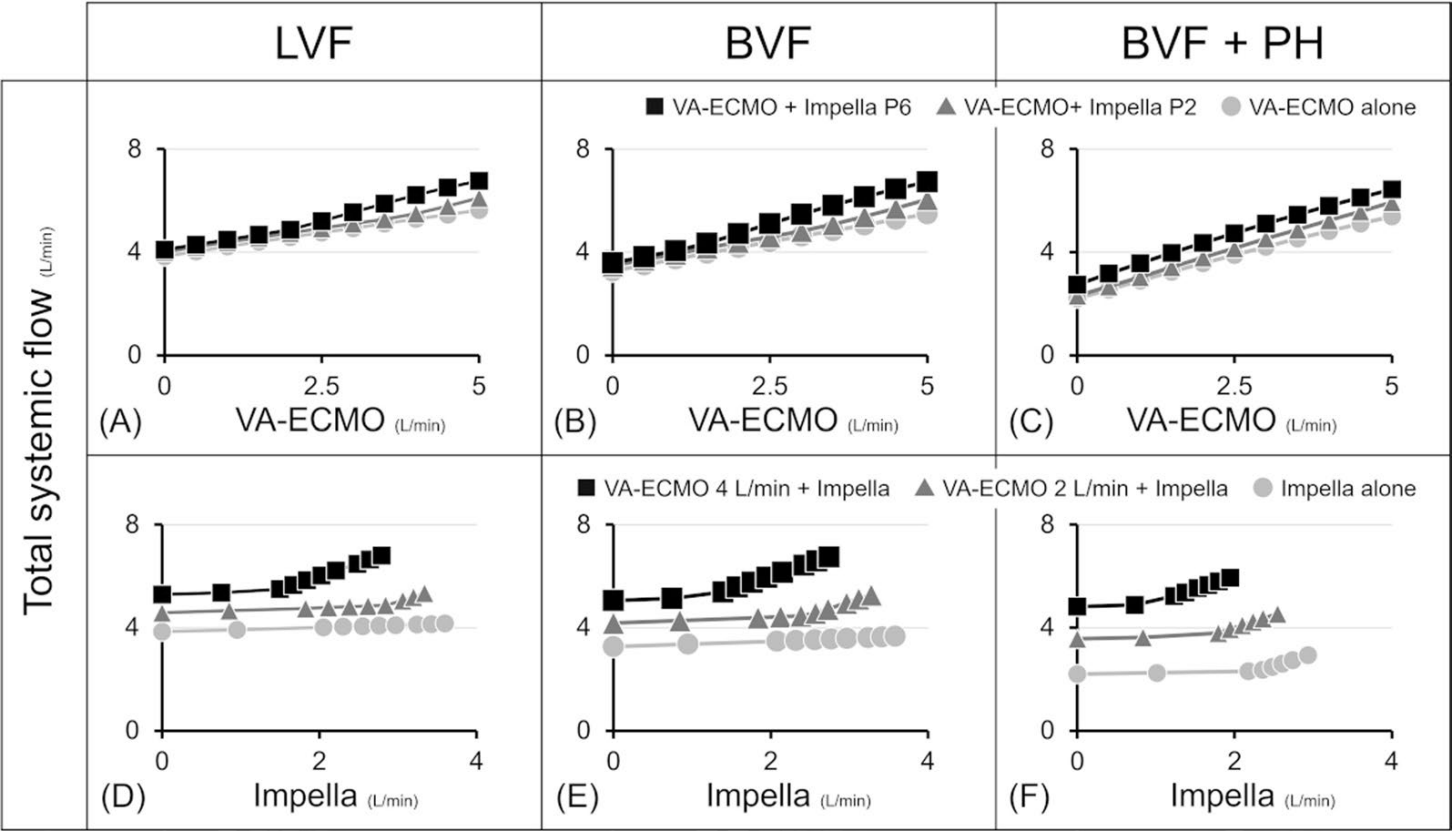
Impact of ECPELLA on total systemic flow. Data from protocols 1–3 (Figs. 3, 5 and 7) were used. Upper panels represent the impact of VA-ECMO flow changes on total systemic flow in LVF (**A**), BVF (**B**), and BVF with PH (**C**), and the comparison among the three conditions: VA-ECMO + Impella P6, VA-ECMO + Impella P2, and VA-ECMO alone. Lower panels represent the impact of Impella support levels on total systemic flow in LVF (**D**), BVF (**E**), and BVF with PH (**F**), and the comparison among the three conditions: VA-ECMO 4 L/min + Impella, VA-ECMO 2 L/min + Impella, and Impella alone. VA-ECMO markedly increased total systemic flow, and the addition of Impella further increased it. The presence of PH significantly limits the flow-supporting effect of VA-ECMO, Impella and ECPELLA. LVF, left ventricular failure; BVF, biventricular failure; PH, pulmonary hypertension; VA-ECMO, veno-arterial extracorporeal membrane oxygenation

We investigated the haemodynamic impacts of VA-ECMO and Impella support levels in various cardiovascular conditions. As shown in Fig. 10, VA-ECMO markedly increased total systemic flow, and the addition of Impella further increased it. Under total ECPELLA support, an increase in Impella support level effectively increased total systemic flow and markedly decreased LAP (Figs. 3, 5 and 7). However, especially in the high PVR condition, an increase in Impella support level excessively decreased LAP and induced LV suction, resulting in limited total systemic flow. ECPELLA has been reported to provide sufficient systemic perfusion with total LV unloading [27] and improve the prognosis for patients through its powerful haemodynamic effects in CS [3]. Impella has been reported to have benefits as an LV venting method for LV distension by VA-ECMO [28]. Our simulation suggested that the efficacy of Impella support in ECPELLA depends on adequate LV filling for stable Impella operation. Optimising the LV filling is difficult because VA-ECMO and PH reduce RV output. Our simulation enables us to understand the optimal haemodynamic management of ECPELLA in several cardiovascular and pump flow conditions.

### The impact of ECPELLA on LV unloading

**Fig. 11:**
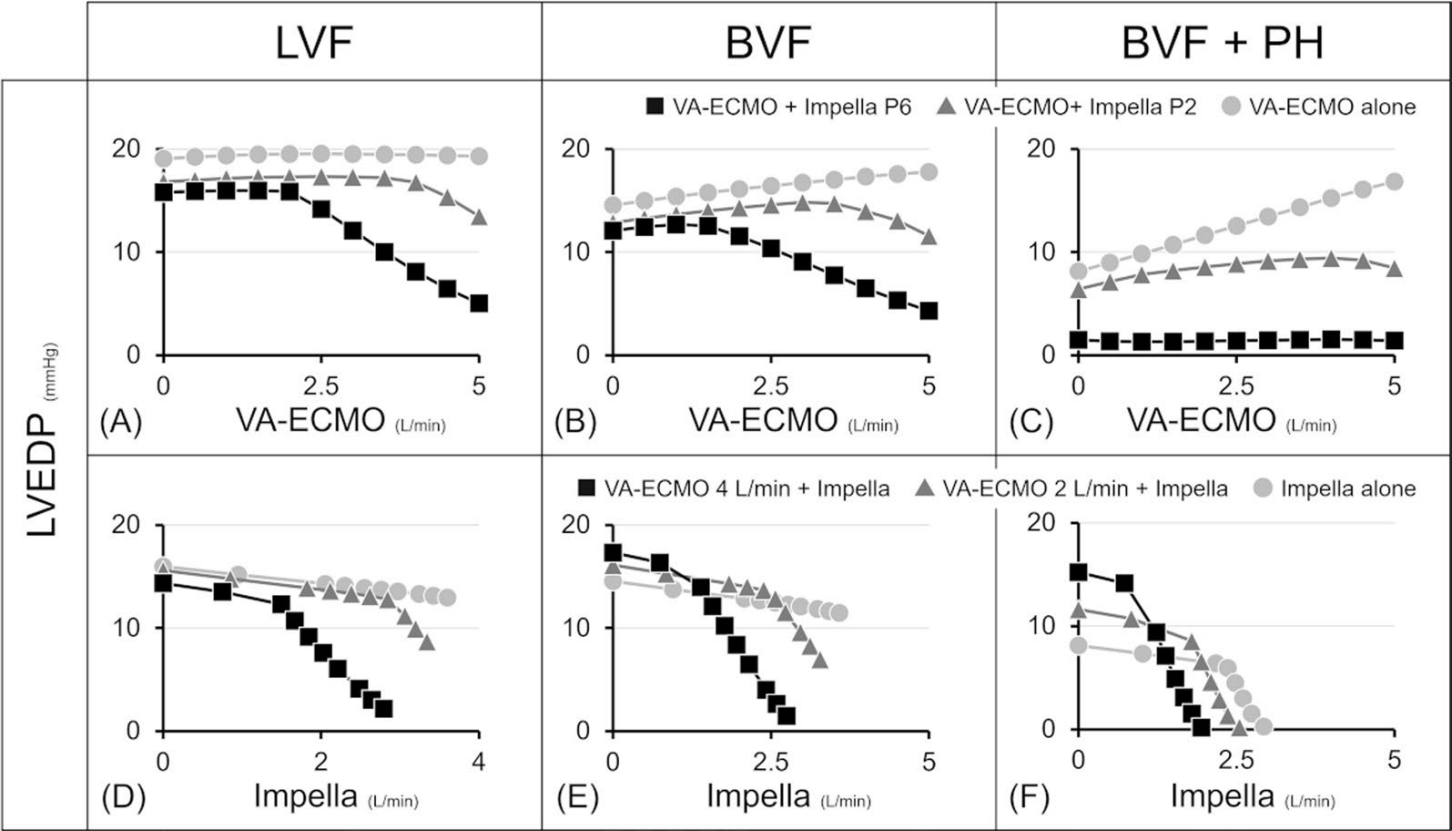
Impact of ECPELLA on LV unloading. Data from protocols 1-3 (Figs. 3, 5 and 7) were used. Upper panels represent the impact of VA-ECMO flow changes on LVEDP, a marker of LV unloading, in LVF (**A**), BVF (**B**), and BVF with PH (**C**), and the comparison among the three conditions: VA-ECMO + Impella P6, VA-ECMO + Impella P2, and VA-ECMO alone. Lower panels represent the impact of Impella support levels on LVEDP in LVF (**D**), BVF (**E**), and BVF with PH (**F**), and the comparison among the three conditions: VA-ECMO 4 L/min + Impella, VAECMO 2 L/min + Impella, and Impella alone. the increase in VA-ECMO flow decreased LVEDP in LVF conditions while increasing it in BVF and BVF with PH conditions. In addition, RV failure enhances the LV unloading effect by ECPELLA. LVF, left ventricular failure; BVF, biventricular failure; PH, pulmonary hypertension; LVEDP, left ventricular end-diastolic pressure; VA-ECMO, veno-arterial extracorporeal membrane oxygenation

The earlier LV unloading can recover LV from acute ischaemic damage [29]. In addition, we also reported that the higher degree of LV unloading by Impella reduces the infarct size in a large animal myocardial infarction model [30]. In this study, we visualised the RV and LV unloading effects of ECPELLA in multiple cardiovascular and pump flow conditions. As shown in Fig. 11, the increase in VA-ECMO flow decreased LV end-diastolic pressure (LVEDP) in LVF conditions while increasing it in BVF and BVF with PH conditions. These findings align with our previous research [31], demonstrating that LV workload can vary depending on the balance between reduced LV preload and elevated LV afterload induced by VA-ECMO. ECPELLA shifted the LV-PV loop downward to the left in each VA-ECMO flow and Impella support level (Figs. 4, 6, 8). In addition, RV failure enhances the LV unloading effect by ECPELLA (Fig. 11). Those changes are also documented in the clinical reports. Unoki et al. reported that ECPELLA provides effective LV unloading and minimises the LP-PV loop in clinical simulation studies of acute myocardial infarction with CS [27]. Bouchez et al. reported that decreased RV function or increased PVR shifted the LV–PV loop downward to the left under left ventricular assist device (LVAD) support [32].

A major advantage of this simulation is the visualisation of the RV-PV loop, which is difficult to estimate in the clinical setting. An increase in the Impella support level shifted the RV-PV loop to the left in the normal PVR conditions (Figs. 4, 6). This indicates a significant decrease in RV afterload and an increase in RV-SV despite an increase in venous return to RV. Since LAP may contribute significantly more than PVR to RV afterload under normal PVR conditions, LV unloading by Impella decreases RV afterload, resulting in increased RV-CO. Meanwhile, when PVR was high, Impella widened the RV–PV loop but did not change RV-PVA and end-systolic pressure, indicating increases in RV preload and RV–SV without a change in RV afterload. Yourshaw et al. reported that Impella progressively improved RAP to pulmonary artery wedge pressure ratio and decreased RV afterload in 25 patients [33]. Farrar et al. reported that LVAD had a beneficial effect in reducing PAP secondary to a reduction in LAP, while it could increase PAP by increasing RV preload when PVR was fixed [34]. Since Impella changes both RV preload and afterload, the impact of Impella on RV workload is complicated. Further simulation studies considering the clinical situation should be conducted.

### The impact of VV-ECMO on global DO_2_ under ECPELLA

In Fig. 9, we showed the impact of VV-ECMO on global DO_2_ in BVF and BVF with PH under ECPELLA. The changes in global DO_2_ closely correspond to the changes in systemic flow in the ECPELLA support condition. Thus, the total support of ECPELLA effectively increased global DO_2_. The elevation of SaO_2_ increased global DO_2_ and augmented the effect of the Impella support level on global DO_2_ elevation. In shock patients, a significant decrease in DO_2_ below the critical level can lead to tissue hypoxia, anaerobic metabolism, organ failure and poor outcomes [35]. Although current clinical guidelines for shock management do not provide specific recommendations for global DO_2_ monitoring, Russell et al. emphasise the importance of monitoring key physiological parameters to optimise DO_2_ [36]. DO_2_-guided strategies in cardiopulmonary bypass management have shown the potential to prevent postoperative acute kidney injury [37]. However, according to Hayes et al., managing high DO_2_ levels may not always be beneficial [38]. Thus, we need to provide individual correction of global DO_2_ in patients with CS.

As shown in Additional file 7: Fig. S5, haemoglobin concentration is a major contributor to global DO_2_ under ECPELLA conditions. On the other hand, for the best support strategy considering the optimal haemoglobin concentration, it is crucial to consider not only the global DO_2_ delivery patterns, but also the local DO_2_ and the oxygen demand of each organ. Additionally, in conditions where PVR is increased due to hypoxemia, increasing venous oxygen saturation (SvO_2_) with VV-ECMO may attenuate hypoxic pulmonary vasoconstriction, reduce PVR, and alter haemodynamics under ECPELLA [39]. Therefore, in a clinical setting, we should also consider the possibility of a more complex effect of VV-ECMO on global DO_2_.

### Approaching clinical knowledge gaps through simulation studies

Despite the positive impact on haemodynamics provided by MCS in CS, the prognosis for survival remains poor [12, 40]. It has been reported that patient background, such as age, plays a significant role in this issue [41]. In addition, the complicated management of multiple MCS may also limit the improvement of outcome in patients with severe CS. Therefore, the strategies and timing of weaning from multiple MCS, including VV-ECMO and VAV-ECMO, are still under debate [42, 43]. Our comprehensive simulation analysis may allow the optimisation of multiple MCS management, including the determination of optimal flow settings and the appropriate timing for weaning from MCS.

### Limitations

This study has several limitations, primarily due to cardiovascular mathematical modelling. First, the simulation did not consider the variability of cardiovascular and respiratory parameters and anatomical and dynamic changes in valve structures. In clinical, various parameters such as LV contractility, heart rate, and vascular resistance can change due to autonomic nerve activity through baroreceptor or cardiopulmonary reflexes [44]. We adopted the normal higher heart rate (80 bpm) based on several reports of CS [12–14]. Meanwhile, Ostadal et al. and Schrage et al. reported higher-than-normal HRs during the acute phase of CS requiring MCS [45, 46]. To develop a more clinically relevant simulator, it is necessary to incorporate the heart rate changes in response to CS condition and its impact of cardiovascular parameters. Increased heart rate can enhance contractility, known as the force-frequency relationship [47]. Furthermore, excessive tachycardia can lead to insufficient relaxation time, potentially causing a reduction in cardiac output, referred to as the incomplete relaxation [48]. Anatomical changes may also affect the haemodynamics in CS with Impella support condition. Mitral and tricuspid valve regurgitation may occur in patients with heart failure [49, 50], and the Impella catheter has been associated with the development or worsening of aortic valve regurgitation [51].

Second, our simulation does not account for VAV-ECMO flow diversion, which can dynamically alter oxygenation, systemic flow and oxygen delivery. In the VAV-ECMO circuit, increased venous outflow may paradoxically decrease arterial outflow due to the inherent limitations of total pump flow. Increasing blood delivery to the arterial side can increase systemic flow and global DO_2_, while compromising regional DO_2_ in vital organs such as the heart and brain, represented by Harlequin syndrome. On the other hand, increasing blood delivery to the venous side can increase SvO_2_ and resolve Harlequin syndrome [52], while it may alter global DO_2_ due to increased SvO_2_, venous cannula recirculation and reduced systemic blood flow. Therefore, future research should focus on developing dynamic models that incorporate ECMO flow diversion and regional DO_2_ distribution to determine the optimal VAV-ECMO flow setting.

Third, in recent years, the importance of integrating micro- and microcirculation management in CS patients has been recognised [53]. Thus, various studies, including methods to assess tissue oxygenation and treatments, are being conducted [54, 55]. Incorporating the effects of tissue oxygenation and microcirculatory changes in the capillary compartment into our simulation is an important next step.

Fourth, our simulation provided a comprehensive understanding of haemodynamic changes under ECPELLA support in various cardiovascular situations. Currently, the simulation aims to help medical staff make optimal decisions by predicting and demonstrating numerous haemodynamic situations under ECPELLA. However, to develop a simulator that provides the best support strategy for each patient individually and instantaneously, it is necessary to automatically estimate each patient's cardiovascular parameters from the haemodynamic monitor. In addition, we need to consider patient-specific oxygen demands in the whole body and vital organs, as well as the individual patient backgrounds such as a causal disease of CS and comorbidities. In the future, the personalised simulator may be able to optimise CS treatments, including drug therapies, transfusions, and MCS settings, which are currently based on clinician experience.

## Conclusions

The optimal ECPELLA support increased total systemic flow and achieved RV and LV unloading. In BVF with severe ARF, the VV-ECMO effectively improves global DO_2_ in total ECPELLA support. Our simulation provides a comprehensive understanding of ECPELLA management in patients with severe CS.

### Supplementary Information

**Additional file 1: **Cardiovascular simulation of ECPELLA haemodynamics.

**Additional file 2: **Head‒capacity (H‒Q) curve of the Impella CP device.

**Additional file 3: **Relationship between the approximate curves and the published H‒Q curves (Instructions for Use and Clinical Reference Manual of Impella CP) at each Impella P level.

**Additional file 4: **Description of pressure-volume area (PVA).

**Additional file 5: **Representative time course of haemodynamic parameters in a haemodynamic simulation of ECPELLA.

**Additional file 6: **Impact of VA-ECMO flow and Impella support level on the right and left ventricular workload in several cardiac functions.

**Additional file 7: **Impact of haemoglobin concentration on global DO2 supported by ECPELLA with VV-ECMO (SaO2: 80%).

## Data Availability

All relevant data obtained and analysed during this study are presented in the main text and the supplement.

## Abbreviations

ARF: Acute respiratory failure
bpm: Beats per minute
BVF: Biventricular failure
CO: Cardiac output
CS: Cardiogenic shock
DO_2_: Oxygen delivery
EDPVR: End-diastolic pressure–volume relationship
*E*_es_: End-systolic elastance
ESPVR: End-systolic pressure–volume relationship
Hb: Haemoglobin concentration
H–Q curve: Head-capacity curve
LAP: Left atrial pressure
LV: Left ventricle
LVAD: Left ventricular assist device
LVEDP: Left ventricular end-diastolic pressure
LVF: Left ventricular failure
MCS: Mechanical circulatory support
PH: Pulmonary hypertension
PV loop: Pressure–volume loop
PVA: Pressure–volume area
PVR: Pulmonary vascular resistance
RAP: Right atrial pressure
RV: Right ventricle
SaO_2_: Arterial oxygen saturation
SvO_2_: Venous oxygen saturation
SVR: Systemic vascular resistance
SW: Stroke work
VA-ECMO: Veno-arterial extracorporeal membrane oxygenation
VV-ECMO: Veno-venous extracorporeal membrane oxygenation
WU: Wood units
Zc: Pulmonary vascular impedance
