# Supplementary material for: The impact of ECPELLA on haemodynamics and global oxygen delivery: a comprehensive simulation of biventricular failure
Source: Intensive Care Med Exp. 2024 Feb 16;12:13. doi: 10.1186/s40635-024-00599-7 (PMC10869331; doi:10.1186/s40635-024-00599-7)
Supplement: Supplementary file 1 — Additional file 1: Cardiovascular simulation of ECPELLA haemodynamics. [file 40635_2024_599_MOESM1_ESM.docx]

**­­Additional file 1: Cardiovascular simulation of ECPELLA haemodynamics**

We have previously developed a cardiovascular simulator (Fig. 1B) using Simulink (Mathworks, Massachusetts, USA) (1). The cardiovascular parameters are defined as follows.

*Modelling of the heart*

Time-varying elastance [E(t)] in each chamber determines the relationship between instantaneous chamber pressure [P(t)] and instantaneous chamber volume [V(t)].

$$\begin{aligned} P\left( t \right)=P_{\mathrm{ed}}\left( V \right)+E\left( t \right)\left[ P_{\mathrm{es}}\left( V \right)-P_{\mathrm{ed}}\left( V \right) \right] \#\left( C1 \right) \end{aligned}$$

in which:

$$\begin{aligned} P_{\mathrm{ed}}\left( V \right)=\alpha{(e}^{\beta\left( V-V_{0} \right)}-1) \#\left( C2 \right) \end{aligned}$$

$$\begin{aligned} P_{\mathrm{es}}\left( V \right)=E_{\mathrm{es}}\left( V-V_{0} \right) \#\left( C3 \right) \end{aligned}$$

and

$$\begin{aligned} E\left( t \right)=\left\{ \begin{matrix} \frac{1}{2}\left\{ \sin\left[ \left( \frac{\pi}{T_{\max}} \right)t-\frac{\pi}{2} \right]+1 \right\} & 0<t\leq\frac{4}{3}T_{\max} \\ \frac{3}{4}e^{-\frac{\left( t-\frac{4}{3}T_{\max} \right)}{\tau}} & t>\frac{4}{3}T_{\max} \end{matrix} \right. \#\left( C4 \right) \end{aligned}$$

where P_ed_(V) is end-diastolic pressure as a function of volume, P_es_(V) is end-systolic pressure as a function of volume, V_0_ is unstressed volume, E_es_ is end-systolic elastance, α and β are constants of the end-diastolic pressure–volume relationship, t is the time from the start of the systole, T_max_ is the point of maximal chamber elastance, and τ is the time constant of relaxation. Intracardiac valves are modelled as unidirectional valves. Parameters of the heart system are fixed as follows.

Parameters of the heart

|  | LA | LV | RA | RV |
| --- | --- | --- | --- | --- |
| E_es_ (mmHg/ml) | 0.5 | 0.4 | 0.2 | 0.2-0.5 |
| α | 3 | 2 | 1.8 | 1 |
| β | 0.025 | 0.01 | 0.025 | 0.02 |
| V_0_ (ml) | 10 | 10 | 10 | 40 |
| T_max_ (s) | 0.15 | 0.3 | 0.15 | 0.3 |
| τ (s) | 0.03 | 0.02 | 0.03 | 0.02 |

LA, left atrium; LV, left ventricle; RA, right atrium; RV, right ventricle; E_es_, end-systolic elastance; V_0_, volume axis; T_max_; time to maximum elastance; τ, time constant.

*Modelling of the vascular system*

Both the systemic and pulmonary vascular systems are modelled using a 5-element resistance–capacitance network model, consisting of arterial, capillary, and venous capacitances (C_A_, C_C_, and C_V_), characteristic impedance (R_Z0_), and arterial, capillary, and venous resistances (R_A_, R_C_, and R_V_). Parameters of the vascular system are fixed as follows.

Parameters of the vascular system

| Compliance (ml/mmHg) | | Resistance (mmHg/ml/s) | | Other parameters | |
| --- | --- | --- | --- | --- | --- |
| C_SA_ | 2.5 | R_SZ0_ | 0.035 | HR (bpm) | 80 |
| C_SC_ | 70 | R_SA_ | 0.7 | SV (ml) | 2500 |
| C_SV_ | 10 | R_SC_ | 0.23 |  |  |
| C_PA_ | 12 | R_SV_ | 0.023 |  |  |
| C_PC_ | 10 | R_PZ0_ | 0.01-0.06 |  |  |
| C_PV_ | 4.5 | R_PA_ | 0.01-0.27 |  |  |
|  |  | R_PC_ | 0.017 |  |  |
|  |  | R_PV_ | 0.01 |  |  |

C_SA_, compliance of systemic artery; C_SC_, compliance of systemic capillary vessels; C_SV_, compliance of systemic vein; C_PA_, compliance of pulmonary artery; C_PC_, compliance of pulmonary capillary vessels; C_PV_, compliance of pulmonary vein; R_SZ0_, characteristic impedance of systemic circulation; R_SA_, resistance of systemic artery; R_SC_, resistance of systemic capillary vessels; R_SV_, resistance of systemic vein; R_PZ0_, characteristic impedance of pulmonary circulation; R_PA_, resistance of pulmonary artery; R_PC_, resistance of pulmonary capillary vessels; R_PV_, resistance of pulmonary vein; HR, heart rate; SV, stressed blood volume.

*Modelling of mechanical circulatory support devices*

For the simulation shown in Figs. 2 to 7 in the main text, we modelled the pump performance of Impella based on the head‒capacity (H‒Q) curve described in a previous report (2) and the company published product manual (Instructions for Use and Clinical Reference Manual of Impella CP). The flow rate of Impella was determined by the Impella rotational speed (P0‒P9) and the pressure gradient between the systemic artery and LV (Additional file 2).

$$\begin{aligned} Q_{\mathrm{Impella}}(P1)=1.57\cdot{10}^{-6}\cdot H_{\mathrm{Impella}}^{4}-1.35\cdot{10}^{-4}\cdot H_{\mathrm{Impella}}^{3} \\ +2.72\cdot{10}^{-3}\cdot H_{\mathrm{Impella}}^{2}-3.32\cdot{{10}^{-2}\cdot H}_{\mathrm{Impella}}+1.84 \#\left( C5 \right) \end{aligned}$$

$$\begin{aligned} Q_{\mathrm{Impella}}\left( P2 \right)=2.36\cdot{10}^{-7}\cdot H_{\mathrm{Impella}}^{4}-4.12\cdot{10}^{-5}\cdot H_{\mathrm{Impella}}^{3} \\ +1.9\cdot{10}^{-3}\cdot H_{\mathrm{Impella}}^{2}-4.29\cdot{{10}^{-2}\cdot H}_{\mathrm{Impella}}+2.66 \#\left( C6 \right) \end{aligned}$$

$$\begin{aligned} Q_{\mathrm{Impella}}\left( P3 \right)=1.06\cdot{10}^{-7}\cdot H_{\mathrm{Impella}}^{4}-2.31\cdot{10}^{-5}\cdot H_{\mathrm{Impella}}^{3} \\ +1.25\cdot{10}^{-3}\cdot H_{\mathrm{Impella}}^{2}-3.53\cdot{{10}^{-2}\cdot H}_{\mathrm{Impella}}+2.81 \#\left( C7 \right) \end{aligned}$$

$$\begin{aligned} Q_{\mathrm{Impella}}\left( P4 \right)=1.34\cdot{10}^{-7}\cdot H_{\mathrm{Impella}}^{4}-2.88\cdot{10}^{-5}\cdot H_{\mathrm{Impella}}^{3} \\ +1.71\cdot{10}^{-3}\cdot H_{\mathrm{Impella}}^{2}-4.60\cdot{{10}^{-2}\cdot H}_{\mathrm{Impella}}+3.04 \#\left( C8 \right) \end{aligned}$$

$$\begin{aligned} Q_{\mathrm{Impella}}\left( P5 \right)=8.36\cdot{10}^{-8}\cdot H_{\mathrm{Impella}}^{4}-2.05\cdot{10}^{-5}\cdot H_{\mathrm{Impella}}^{3} \\ +1.37\cdot{10}^{-3}\cdot H_{\mathrm{Impella}}^{2}-4.25\cdot{{10}^{-2}\cdot H}_{\mathrm{Impella}}+3.23 \#\left( C9 \right) \end{aligned}$$

$$\begin{aligned} Q_{\mathrm{Impella}}\left( P6 \right)=5.74\cdot{10}^{-8}\cdot H_{\mathrm{Impella}}^{4}-1.59\cdot{10}^{-5}\cdot H_{\mathrm{Impella}}^{3} \\ +1.20\cdot{10}^{-3}\cdot H_{\mathrm{Impella}}^{2}-4.12\cdot{{10}^{-2}\cdot H}_{\mathrm{Impella}}+3.42 \#\left( C10 \right) \end{aligned}$$

$$\begin{aligned} Q_{\mathrm{Impella}}\left( P7 \right)=2.94\cdot{10}^{-8}\cdot H_{\mathrm{Impella}}^{4}-9.83\cdot{10}^{-6}\cdot H_{\mathrm{Impella}}^{3} \\ +3.82\cdot{10}^{-4}\cdot H_{\mathrm{Impella}}^{2}-3.65\cdot{{10}^{-2}\cdot H}_{\mathrm{Impella}}+3.68 \#\left( C11 \right) \end{aligned}$$

$$\begin{aligned} Q_{\mathrm{Impella}}\left( P8 \right)=1.98\cdot{10}^{-8}\cdot H_{\mathrm{Impella}}^{4}-7.44\cdot{10}^{-6}\cdot H_{\mathrm{Impella}}^{3} \\ +7.40\cdot{10}^{-4}\cdot H_{\mathrm{Impella}}^{2}-3.42\cdot{{10}^{-2}\cdot H}_{\mathrm{Impella}}+3.87 \#\left( C12 \right) \end{aligned}$$

$$\begin{aligned} Q_{\mathrm{Impella}}\left( P9 \right)=1.78\cdot{10}^{-8}\cdot H_{\mathrm{Impella}}^{4}-6.97\cdot{10}^{-6}\cdot H_{\mathrm{Impella}}^{3} \\ +7.43\cdot{10}^{-4}\cdot H_{\mathrm{Impella}}^{2}-3.56\cdot{{10}^{-2}\cdot H}_{\mathrm{Impella}}+4.05 \#\left( C13 \right) \end{aligned}$$

where Q_Impella_ is Impella flow, H_Impella_ is the pressure difference between LV and aorta. The approximate H-Q curves (P0-P9) correlate significantly (p < 0.05, Spearman's rank correlation coefficient) with the published H-Q curves (Additional file 2). Veno-arterial ECMO (VA-ECMO) was designed to continuously remove blood from the systemic vein and return to the systemic artery at continuous flow.

1. Saku K, Kakino T, Arimura T, Sunagawa G, Nishikawa T, Sakamoto T, et al. Left Ventricular Mechanical Unloading by Total Support of Impella in Myocardial Infarction Reduces Infarct Size, Preserves Left Ventricular Function, and Prevents Subsequent Heart Failure in Dogs. Circ Heart Fail. 2018 May;11(5):e004397.
2. Unoki T, Saku K, Kametani M, Konami Y, Taguchi E, Sawamura T, et al. Impella Motor Current Amplitude Reflects the Degree of Left Ventricular Unloading under ECPELLA Support. Int Heart J. 2022;63(6):1187-93.
