# Supplementary material for: The impact of ECPELLA on haemodynamics and global oxygen delivery: a comprehensive simulation of biventricular failure
Source: Intensive Care Med Exp. 2024 Feb 16;12:13. doi: 10.1186/s40635-024-00599-7 (PMC10869331; doi:10.1186/s40635-024-00599-7)
Supplement: Supplementary file 2 — Additional file 2: Head‒capacity (H‒Q) curve of the Impella CP device. [file 40635_2024_599_MOESM2_ESM.docx]

**­­Additional file 2: Head‒capacity (H‒Q) curve of the Impella CP device**

**
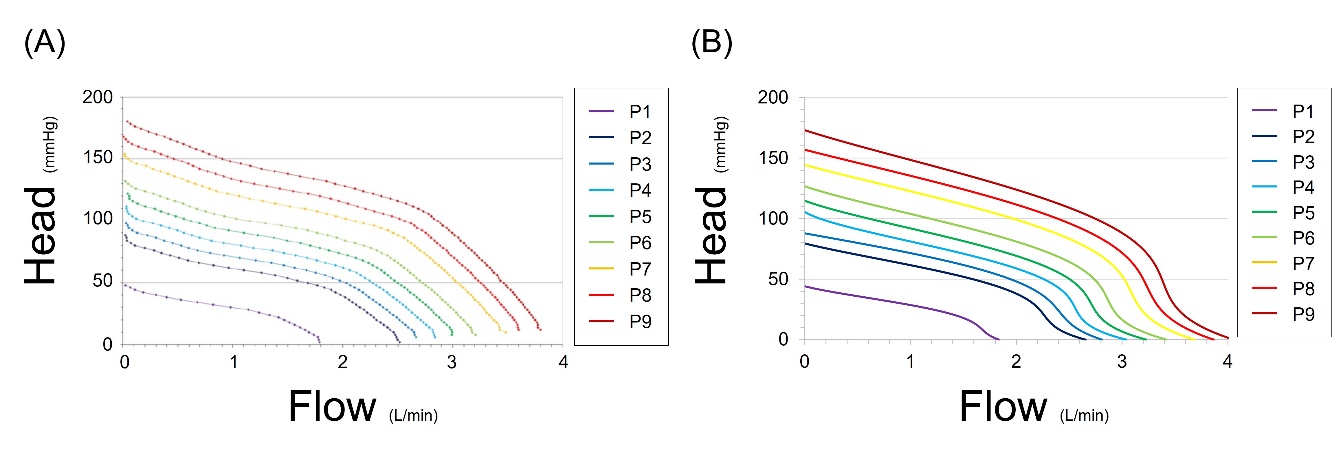
**

Head‒capacity (H‒Q) curve of the Impella CP® device (Abiomed, Inc, Danvers, USA). (A) The published H-Q curve of the Impella CP® device in each the P level (Instructions for Use and Clinical Reference Manual of Impella CP). (B) Approximate H‒Q curve of the Impella CP. The flow rate of Impella was determined by the Impella rotational speed (P0‒P9) and the pressure gradient between the systemic artery and the left ventricle. Formulas for approximate H-Q curves at each support level incorporated in the simulation are described in Additional file 1.
