# Supplementary material for: The impact of ECPELLA on haemodynamics and global oxygen delivery: a comprehensive simulation of biventricular failure
Source: Intensive Care Med Exp. 2024 Feb 16;12:13. doi: 10.1186/s40635-024-00599-7 (PMC10869331; doi:10.1186/s40635-024-00599-7)
Supplement: Supplementary file 3 — Additional file 3: Relationship between the approximate curves and the published H‒Q curves (Instructions for Use and Clinical Reference Manual of Impella CP) at each Impella P level. [file 40635_2024_599_MOESM3_ESM.docx]

**­­Additional file 3: Relationship between the approximate curves and the published H‒Q curves (Instructions for Use and Clinical Reference Manual of Impella CP) at each Impella P level**

**
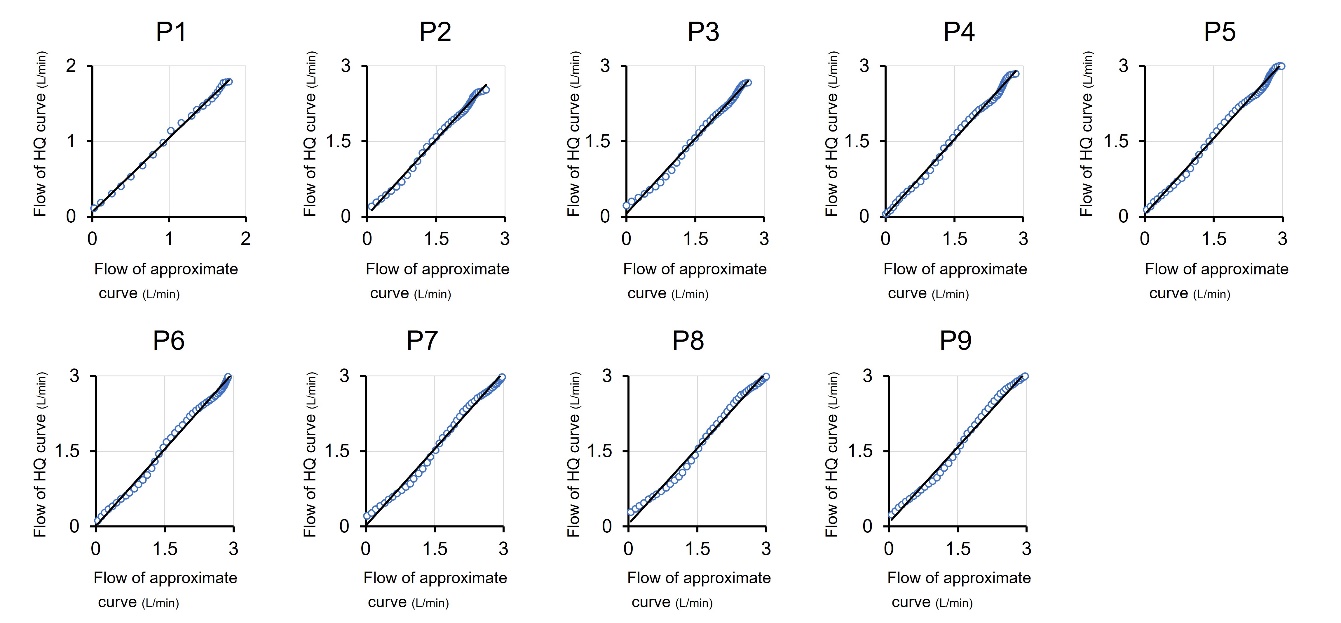
**

Relationship between the approximate curves and the published H‒Q curves (Instructions for Use and Clinical Reference Manual of Impella CP) at each Impella P level. The approximate curves are compared with the published H-Q curves by performing statistical analysis using Spearman's rank correlation coefficient. All the approximate curves correlate significantly (p < 0.05) with the published H-Q curves.
