# Supplementary material for: The impact of ECPELLA on haemodynamics and global oxygen delivery: a comprehensive simulation of biventricular failure
Source: Intensive Care Med Exp. 2024 Feb 16;12:13. doi: 10.1186/s40635-024-00599-7 (PMC10869331; doi:10.1186/s40635-024-00599-7)
Supplement: Supplementary file 4 — Additional file 4: Description of pressure-volume area (PVA). [file 40635_2024_599_MOESM4_ESM.docx]

**­­Additional file 4: Description of pressure-volume area (PVA)**

**
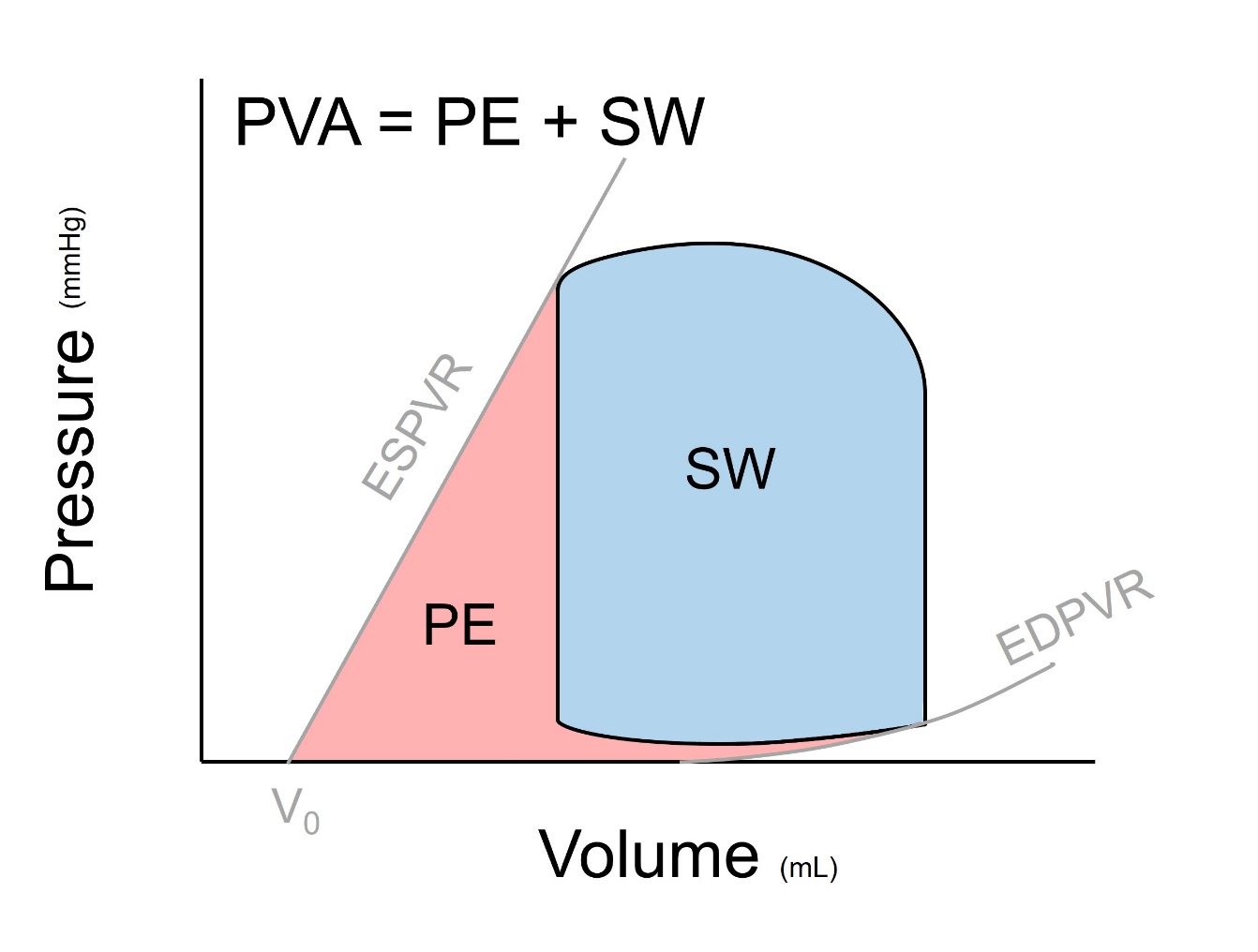
**

The pressure-volume area (PVA) was calculated by integrating the area enclosed by the end-systolic pressure-volume relationship (ESPVR), the end-diastolic pressure‒volume relationship (EDPVR), and the PV loop. Pressure-volume area (PVA) is the sum of the stroke work (SW) and potential energy (PE).
