# Supplementary material for: The impact of ECPELLA on haemodynamics and global oxygen delivery: a comprehensive simulation of biventricular failure
Source: Intensive Care Med Exp. 2024 Feb 16;12:13. doi: 10.1186/s40635-024-00599-7 (PMC10869331; doi:10.1186/s40635-024-00599-7)
Supplement: Supplementary file 5 — Additional file 5: Representative time course of haemodynamic parameters in a haemodynamic simulation of ECPELLA. [file 40635_2024_599_MOESM5_ESM.docx]

**­­Additional file 5: Representative time course of haemodynamic parameters in a haemodynamic simulation of ECPELLA**

**
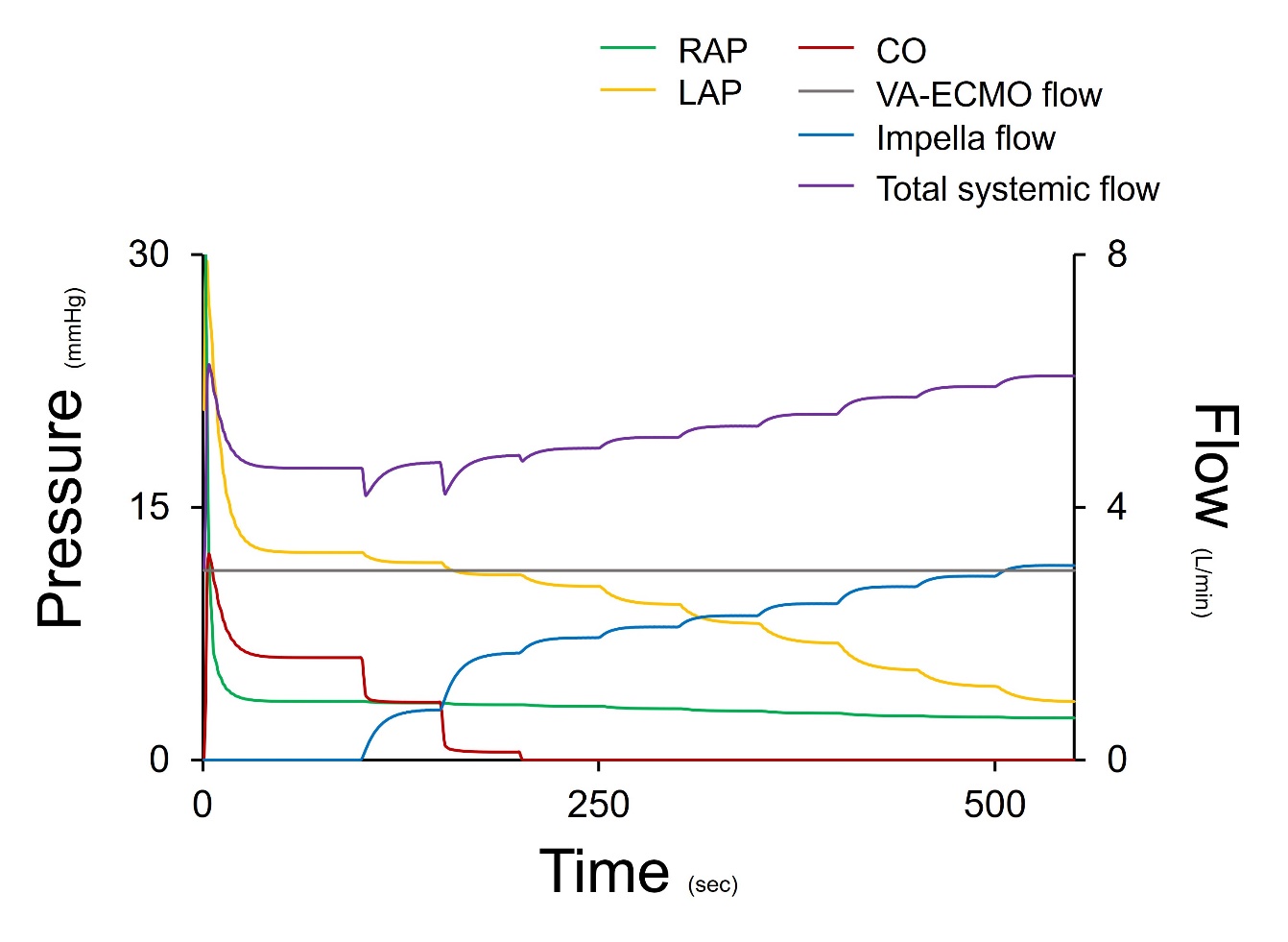
**

Representative time course of haemodynamic parameters from one cardiovascular simulation. For the first 100 seconds, haemodynamic simulations were conducted with VA-ECMO and without Impella support. Then, the Impella support level was increased gradually every 50 seconds, and stable haemodynamic values were extracted 2 seconds before the next alteration when the time series data reached a steady state. RAP, right atrial pressure; LAP, left atrial pressure; CO, cardiac output; VA-ECMO, veno-arterial extracorporeal membrane oxygenation.
