# Supplementary material for: The impact of ECPELLA on haemodynamics and global oxygen delivery: a comprehensive simulation of biventricular failure
Source: Intensive Care Med Exp. 2024 Feb 16;12:13. doi: 10.1186/s40635-024-00599-7 (PMC10869331; doi:10.1186/s40635-024-00599-7)
Supplement: Supplementary file 6 — Additional file 6: Impact of VA-ECMO flow and Impella support level on the right and left ventricular workload in several cardiac functions. [file 40635_2024_599_MOESM6_ESM.docx]

**Additional file 6: Impact of VA-ECMO flow and Impella support level on the right and left ventricular workload in several cardiac functions**

Impact of VA-ECMO flow and Impella support level on the right and left ventricular workload in LVF haemodynamics

| VA-ECMO | PVA / SW | | Baseline | Impella P2 | Impella P6 |
| --- | --- | --- | --- | --- | --- |
| (L/min) | (mmHg･mL･10^3^) | |  |  |  |
| none | RV | PVA | 1.27 | 1.19 | 1.15 |
|  |  | SW | 0.90 | 0.88 | 0.86 |
|  | LV | PVA | 8.67 | 7.72 | 7.21 |
|  |  | SW | 2.82 | 2.19 | 1.86 |
| 2.0 | RV | PVA | 0.88 | 0.82 | 0.78 |
|  |  | SW | 0.57 | 0.56 | 0.55 |
|  | LV | PVA | 9.04 | 8.03 | 7.29 |
|  |  | SW | 2.10 | 1.45 | 1.03 |
| 4.0 | RV | PVA | 0.47 | 0.43 | 0.30 |
|  |  | SW | 0.25 | 0.26 | 0.23 |
|  | LV | PVA | 9.10 | 7.93 | 4.19 |
|  |  | SW | 1.13 | 0.57 | 0.62 |

VA-ECMO, veno-arterial extracorporeal membrane oxygenation; LVF, left ventricular failure; RV, right ventricle; LV, left ventricle; PVA, pressure‒volume area; SW, stroke work.

Impact of VA-ECMO flow and Impella support level on the right and left ventricular workload in BVF haemodynamics

| VA-ECMO | PVA / SW | | Baseline | Impella P2 | Impella P6 |
| --- | --- | --- | --- | --- | --- |
| (L/min) | (mmHg･mL･10^3^) | |  |  |  |
| None | RV | PVA | 0.79 | 0.75 | 0.73 |
|  |  | SW | 0.37 | 0.38 | 0.38 |
|  | LV | PVA | 7.25 | 6.32 | 5.84 |
|  |  | SW | 2.28 | 1.66 | 1.36 |
| 2.0 | RV | PVA | 0.69 | 0.65 | 0.58 |
|  |  | SW | 0.28 | 0.29 | 0.30 |
|  | LV | PVA | 7.99 | 6.99 | 5.67 |
|  |  | SW | 1.72 | 1.08 | 0.87 |
| 4.0 | RV | PVA | 0.49 | 0.42 | 0.26 |
|  |  | SW | 0.14 | 0.16 | 0.16 |
|  | LV | PVA | 8.43 | 6.95 | 3.35 |
|  |  | SW | 0.91 | 0.50 | 0.54 |

VA-ECMO, veno-arterial extracorporeal membrane oxygenation; BVF, biventricular failure; RV, right ventricle; LV, left ventricle; PVA, pressure‒volume area; SW, stroke work.

Impact of VA-ECMO flow and Impella support level on the right and left ventricular workload in BVF with PH haemodynamics

| VA-ECMO | PVA / SW | | Baseline | Impella P2 | Impella P6 |
| --- | --- | --- | --- | --- | --- |
| (L/min) | (mmHg･mL･10^3^) | |  |  |  |
| None | RV | PVA | 0.96 | 0.95 | 0.94 |
|  |  | SW | 0.28 | 0.29 | 0.31 |
|  | LV | PVA | 4.52 | 3.29 | 0.41 |
|  |  | SW | 1.28 | 0.62 | 0.21 |
| 2.0 | RV | PVA | 0.90 | 0.89 | 0.87 |
|  |  | SW | 0.24 | 0.26 | 0.31 |
|  | LV | PVA | 6.28 | 4.50 | 0.36 |
|  |  | SW | 1.15 | 0.52 | 0.17 |
| 4.0 | RV | PVA | 0.66 | 0.63 | 0.64 |
|  |  | SW | 0.12 | 0.17 | 0.23 |
|  | LV | PVA | 7.71 | 5.03 | 0.47 |
|  |  | SW | 0.69 | 0.38 | 0.16 |

VA-ECMO, veno-arterial extracorporeal membrane oxygenation; BVF, biventricular failure; PH, pulmonary hypertension; RV, right ventricle; LV, left ventricle; PVA, pressure-volume area; SW, stroke work.
