# Supplementary material for: The impact of ECPELLA on haemodynamics and global oxygen delivery: a comprehensive simulation of biventricular failure
Source: Intensive Care Med Exp. 2024 Feb 16;12:13. doi: 10.1186/s40635-024-00599-7 (PMC10869331; doi:10.1186/s40635-024-00599-7)
Supplement: Supplementary file 7 — Additional file 7: Impact of haemoglobin concentration on global DO2 supported by ECPELLA with VV-ECMO (SaO2: 80%). [file 40635_2024_599_MOESM7_ESM.docx]

**Additional file 7: Impact of haemoglobin concentration on global DO_2_ supported by ECPELLA with VV-ECMO (SaO_2_: 80%)**

**
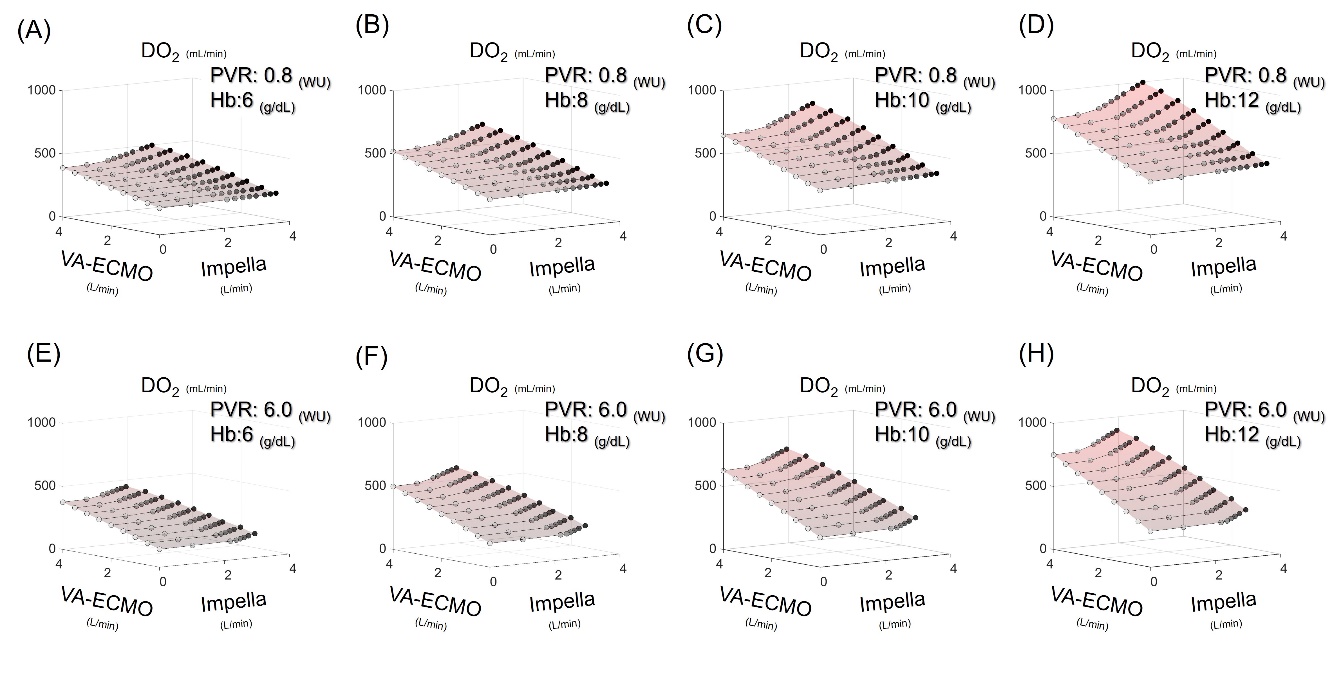
**

Impact of haemoglobin concentration on global DO_2_ supported by ECPELLA with VV-ECMO (SaO_2_: 80%). Upper panels illustrate global DO_2_ changes at various haemoglobin concentrations: 6 g/dL (A), 8 g/dL (B), 10 g/dL (C), and 12 g/dL (D) in BVF. Lower panels depict global DO_2_ changes at the same haemoglobin levels: 6 g/dL (E), 8 g/dL (F), 10 g/dL (G), and 12 g/dL (H) in BVF with PH. Lower haemoglobin concentrations diminish the effectiveness of both VA-ECMO and Impella in increasing global DO_2_. DO_2_, oxygen delivery; VV-ECMO, veno-venous membrane oxygenation; VA-ECMO, veno-arterial extracorporeal membrane oxygenation; SaO_2_, arterial oxygen saturation.
